# Phylogenetic systematics of the genera of Thryptocerina Jeannel, 1949 and new species from New Caledonia (Coleoptera, Carabidae, Oodini)

**DOI:** 10.3897/zookeys.1044.63775

**Published:** 2021-06-16

**Authors:** Kipling Will, Borislav Guéorguiev

**Affiliations:** 1 University of California Berkeley, Essig Museum of Entomology. Berkeley, California, USA University of California Berkeley United States of America; 2 National Museum of Natural History, 1 Blvd. Tsar Osvoboditel, 1000 Sofia, Bulgaria National Museum of Natural History Sofia Bulgaria

**Keywords:** Cladistic analysis, morphology, revisionary systematics

## Abstract

The Oodini precinctive to New Caledonia are reviewed with nine species recognized, of which seven are newly described in two genera. Five species are described in the genus *Coptocarpus* Chaudoir: *C. microps***sp. nov.**, *C. erwini***sp. nov.**, *C. amieuensis***sp. nov.**, *C. magnus***sp. nov.**, and *C. lescheni***sp. nov.** In the genus *Adelopomorpha* Heller two species, *A. tethys***sp. nov.** and *A. tuberculata***sp. nov.**, are described. In order to place cladistically the newly described species in a genus, a phylogenetic analysis of a matrix of 36 characters of adult morphology was conducted including exemplar species of three putative outgroup genera, six putative ingroup thryptocerine oodine genera, and all oodine species from New Caledonia. Results show support for Thryptocerina and monophyly of *Adelopomorpha*. *Hoplolenus* LaFerté-Sénectère is not monophyletic and *Hoplolenus cyllodinus* Fauvel is newly combined as *Coptocarpus cyllodinus***comb. nov.** New Caledonian species of *Coptocarpus* form a clade, but the Australian species of the genus included in the analysis are rendered paraphyletic by African and Malagasy genera. Implications of this preliminary study for the classification of Oodini and trends in the evolution of the female reproductive tract are discussed. A key to the New Caledonian species of Oodini is provided.

## Introduction

Terry Erwin’s extensive knowledge of carabids worldwide is what allowed him to contribute greatly to our understanding of the family’s evolution (Erwin 1985) and classification (Erwin 1985, 1991b). In terms of taxonomic and faunistic coverage, his publications on carabid beetles were almost exclusively focused on New World taxa, particularly the Neotropical fauna. Armed with empirical data, largely based on carabid beetles from the Neotropics, Erwin was an early champion of the cause of conservation worldwide and frequently sounded a clarion call to discover, describe, and conserve invertebrates, and to value the planet’s biological rich habitats (e.g., Erwin 1982, 1991a; Erwin and Geraci 1985; Lucky et al. 1992; Cardoso et al. 2011; Erwin et al. 2012). While his passion for the Neotropics was clearly evident, he did on occasion treat taxa outside of the region. For example, his 1974 revision of Australian *Coptocarpus* Chaudoir, 1857 (Oodini) and his treatment of the carabid fauna of Sri Lanka (Erwin 1984). Herein we intend to honor Terry Erwin’s legacy by treating unrevised *Coptocarpus* and *Adelopomorpha* Heller, 1916 from the smallest, but arguably one of the most significant and threatened biodiversity hotspots (Wulff et al. 2013), New Caledonia.

Early works covering a significant number of New Caledonian carabid species were published during the time that European exploration and colonization across the Pacific was in its heyday (e.g., Perroud and Montrouzier 1864; Fauvel 1882, 1903; Heller 1916). There are scattered works on the carabids of New Caledonia over the following decades and then a notable uptick in new species described in the last 10 years, with nearly 40% of the around 200 species recorded from the island being described in this most recent period. Significant additions to the fauna include new species in Odacanthini (Liebherr 2016), Moriomorphini (Liebherr 2018), Trechini (Donabauer 2011; Giachino et al 2012; Liebherr 2020), Dyschiriini (Bulirsch 2010), Broscini (Seldon and Holwell 2019), Moriomorphini (Liebherr 2018), Chlaeniini (Kirschenhofer 2015), Harpalini (Jaeger 2016), Pterostichini (Will 2011), and Abacetini (Will 2020a). Arguably two factors greatly contributed to the recent increase in descriptive publications, 1) a reinvigorated interest in New Caledonian biogeography due to a controversial proposal that the main island, Grande Terre, was submerged until possibly as late as the late Eocene (Grandcolas et al. 2008, but see Maurizot and Campbell 2020) and, most significantly, 2) major invertebrate sampling efforts by the Queensland Museum in the early 2000s that generated a large amount of scientifically important specimens (Monteith et al. 2005, 2006), including the bulk of the oodine specimens used in this study.

In the Australasian Region, representatives of the tribe Oodini LaFerté-Sénectère, 1851 are distributed in Australia (27 species in *Anatrichis* LeConte, 1853; *Coptocarpus*; *Nanodiodes* Bousquet, 1996; and *Oodes* Bonelli, 1810), New Guinea (14 species in *Anatrichis*; *Brachyodes* Jeannel, 1949; *Coptocarpus*; *Nanodiodes*; *Oodes*; and *Oodinus* Motschulsky, 1864), and New Caledonia (by one species each of *Hoplolenus* LaFerté-Sénectère, 1851 and *Adelopomorpha* prior to this study), with Australian *Coptocarpus* being the only genus in the Australasian Region that has been more or less recently reviewed (Erwin 1974; Baehr 2017).

Albert Fauvel (1882) described the first New Caledonian representative of the tribe, *Hoplolenus cyllodinus* Fauvel, 1882 from a single male specimen. Fauvel noted that this species possesses all the principal (generic) characters listed by Chaudoir (1857) for *Hoplolenus insignis* LaFerté-Sénectère, 1851. At the undertaking of this study, *Hoplolenus* included three Afrotropical and Fauvel’s New Caledonian species (Lorenz 2005).

Early in the 20^th^ century, Heller (1916) discovered a second specimen of Oodini from New Caledonia. Based on a single female, this author proposed the genus *Adelopomorpha* to accommodate the single species *A. glabra* Heller, 1916. Heller listed character states by which his species differs from representatives of *Hoplolenus*: the maxillary palpomere with terminal article longer than the penultimate one; the six-punctate labrum; the long, stretched mandible; the lanceolate prosternal process embedded in a deep, sharply raised groove of the mesosternum; and the elytral striae completely obliterated apically. *Adelopomorpha* has since remained a monotypic genus (Lorenz 2005).

**Figures 1–4.**
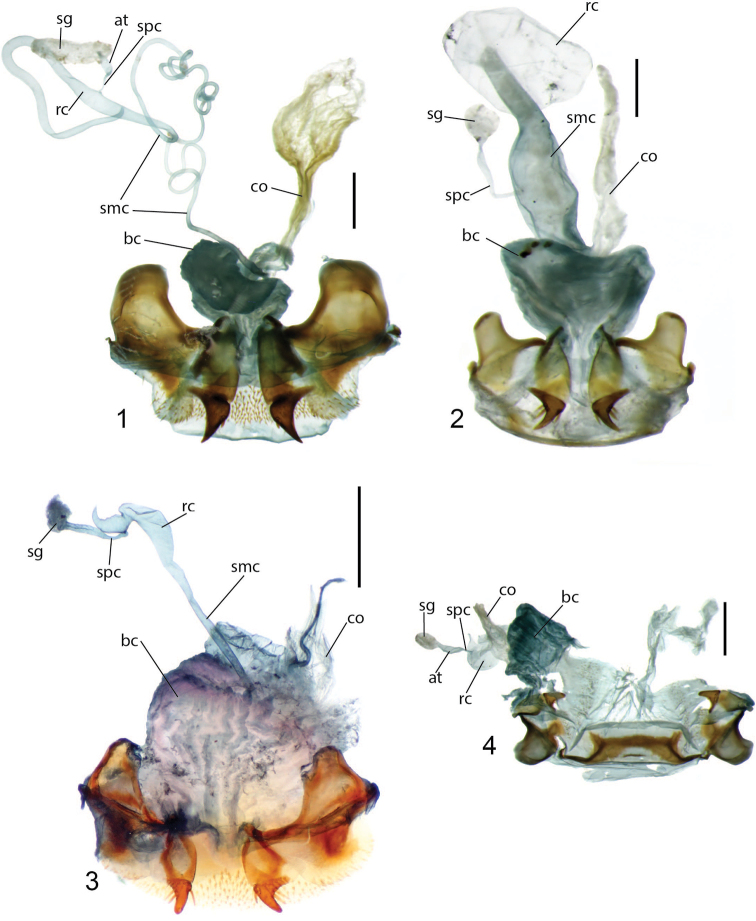
Female reproductive tract ventral view of **1***Simous annamita* Csiki (Laos) **2***Lobatodes decellei* Basilewsky (Ibadan, Nigeria) **3***Holcocoleus latus* LaFerté-Sénectère (Coimbatore, India) **4***H. melanopus* Andrewes (Madras, India). Abbreviations: at, atrium; bc, bursa copulatrix; co, common oviduct; rc, receptaculum; sg, spermatheca gland; smc, seminal canal; spc, spermathecal canal. Scale bars: 0.5 mm.

The genus-level taxonomy of Oodini is in need of revision though there have been some significant recent advances (Bousquet 1996; Guéorguiev 2014; Guéorguiev and Liang 2020). While any generic arrangement will be somewhat provisional given the absence of a worldwide treatment, the placement of the previously described New Caledonian species and those described herein did not obviously align with the characteristics of *Hoplolenus* and *Adelopomorpha*, at least not without consideration of the broader set of genera likely to be close relatives. Several genera, including *Hoplolenus*, *Coptocarpus*, *Thryptocerus* Chaudoir, and *Orthocerodus* Basilewsky, have long been proposed to be closely related genera in “Thryptocerini” of Jeannel (1949), an appellation proposed for those oodine carabids with basal bulb of the median lobe of aedeagus relatively large, convex, with a smooth and rounded dorsal surface. Indeed, as discussed by Erwin (1974) in his revision of *Coptocarpus*, all of these genera and *Adelopomorpha* share many significant characteristics that likely include a number of synapomorphies for the group. However, aside from the single New Caledonian species placed in *Hoplolenus*, only *Coptocarpus* and *Adelopomorpha* are found in the Australian region. All others are restricted to Madagascar (*Thryptocerus* and *Orthocerodus*) or western central Africa (three species of *Hoplolenus*). Preliminary data suggest that representatives of the Afrotropical *Lobatodes* Basilewsky, 1956, also belong to this lineage. Characters that are shared by these are pronotum lacking setiferous punctures near the hind angles and abdominal ventrites 1–5 without ambulatory setae (except *Coptocarpus erwini* sp. nov.). In addition, all genera except *Adelopomorpha* have male protarsomeres 1–3 strongly dilated and 2–4 eccentrically attached to the preceding tarsomere (with basal axis of the former affixed on lateral 1/2–1/3 of the latter) as well as the basal 1/3 or more of the ventral surface of male protarsomere 1 glabrous, i.e., lacking squamose setae.

**Figures 5–8.**
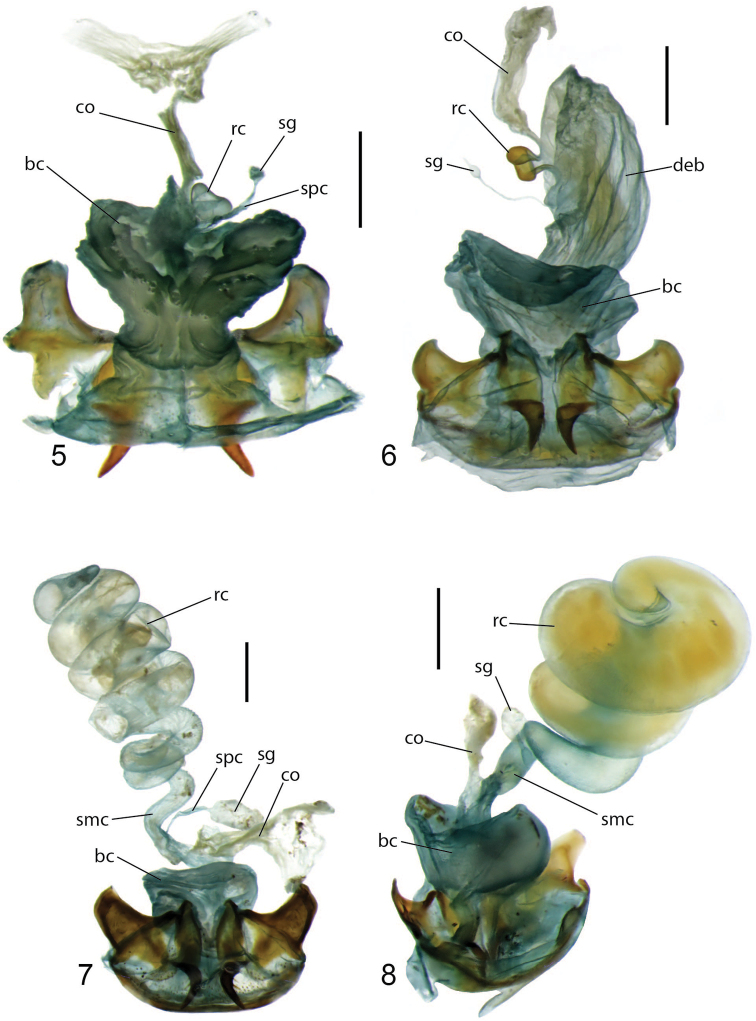
Female reproductive tract of **5***Hoplolenus insignis* LaFerté-Sénectère (dorsal view, Liberia), **6***Thryptocerus agaboides* (Fairmaire) (ventral view, Imerina, Madagascar) **7***Coptocarpus* sp. *cf. chaudoiri* (ventral view, Blackbull Creek, Queensland, Australia) **8***C. thoracicus* (Laporte) (dorsal view, Frankland River, Western Australia, Australia). Abbreviations: at, atrium; bc, bursa copulatrix; co, common oviduct; deb, distal extension of bursa copulatrix; rc, receptaculum; sg, spermatheca gland; smc, seminal canal; spc, spermathecal canal. Scale bars: 0.5 mm.

**Figures 9–12.**
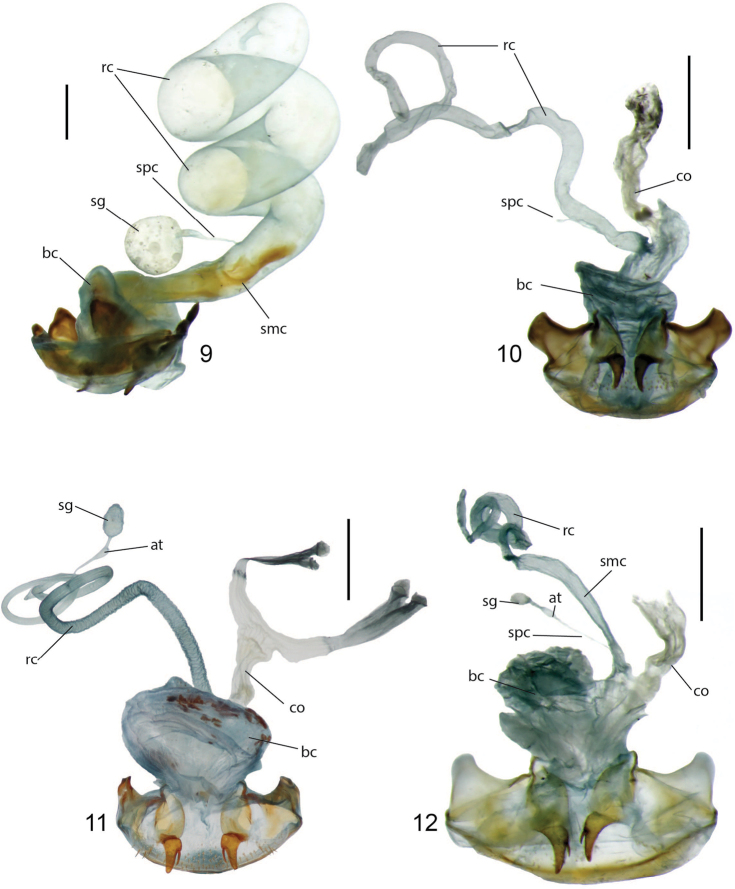
Female reproductive tract of **9***Coptocarpus grossus* Erwin (dorsal view, Margaret River, Western Australia, Australia) **10***Coptocarpus* sp. group 6 (ventral view, Mt. Lewis, Queensland, Australia) **11***C. erwini* sp. nov. (ventral view, Mt. Humboldt, New Caledonia) **12***C. cyllodinus* (Fauvel) (ventral view, Riviére Bleue, New Caledonia). Abbreviations: at, atrium; bc, bursa copulatrix; co, common oviduct; rc, receptaculum; sg, spermatheca gland; smc, seminal canal; spc, spermathecal canal. Scale bars: 0.5 mm.

**Figures 13–16.**
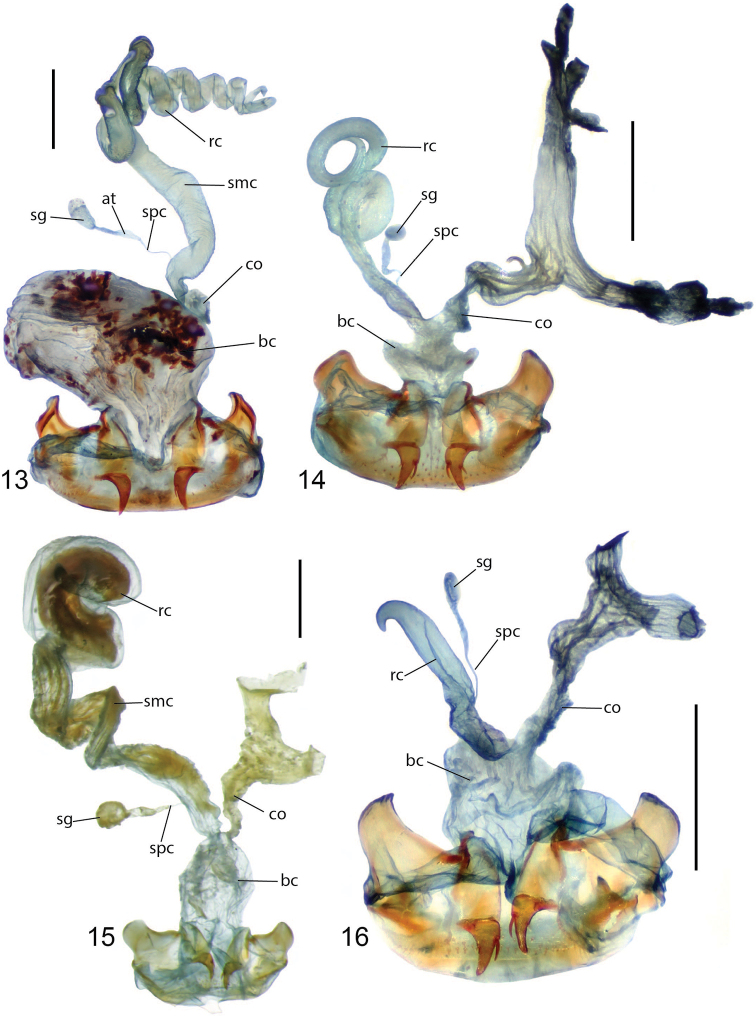
Female reproductive tract, ventral view of New Caledonian species **13***Coptocarpus magnus* sp. nov. (Me Maoya Camp) **14***Adelopomorpha glabra* Heller (Mt. Panie) **15***glabra* group type 1 female (Mt. Koghis) **16***glabra* group type 2 female (Me Maoya Camp). Abbreviations: at, atrium; bc, bursa copulatrix; co, common oviduct; rc, receptaculum; sg, spermatheca gland; smc, seminal canal; spc, spermathecal canal. Scale bars: 0.5 mm.

However, several morphological characters suggest a possible misalignment of the New Caledonian species. Notably the three Afrotropical *Hoplolenus* and representatives of *Thryptocerus* and *Orthocerodus* have the middle four setae of the labrum grouped in a central depression and isolated from the foveae of the lateral two setae, a characteristic not found in *Lobatodes* and Australasian “Thryptocerini.” Also, members of Afrotropical *Hoplolenus*, *Thryptocerus* and *Orthocerodus* lack elytral discal punctures on interval 3, whereas such punctures are present in *Lobatodes*, and variably present or absent within *Coptocarpus*. In addition, members of the Afrotropical *Hoplolenus* lack the lateral bead of the pronotum and have a very short, semi-spherical receptaculum (Fig. 5) (sessile form). All *Coptocarpus* have the setae of the labrum in independent depressions, or in one case (*C. lescheni* sp. nov.) the labrum has only four setae with two medially grouped; the pronotum has a complete, well-marked indication of the lateral bead; and the receptaculum is elongate (e.g., Figs 7–13).

Given the initial uncertainty of generic placement for the obviously undescribed, species-level diversity we found in samples of oodines from New Caledonia, we conducted a phylogenetic analysis of a suite of morphological characters, including those noted above and others, and used the resultant tree to establish the classification and cladistic circumscription for the New Caledonian species. Additionally, this study builds on Erwin’s discussion of *Coptocarpus* and putatively related genera and is a significant step forward in understanding the relationships of thryptocerine oodines.

## Materials and methods

### Morphological methods

A total of 46 specimens of New Caledonian oodines was examined from the following collections for this study:

**EMEC**Essig Museum of Entomology, Berkeley, California, USA (Kipling Will);

**HNHM**Hungarian Natural History Museum, Budapest, Hungary (Ottó Merkl†, Győző Szél);

**MNHN** Muséum National d’Histoire Naturelle, Paris (Thierry Deuve, Azadeh Taghavian);

**MTKD**Museum für Tierkunde, Dresden, Germany (Olaf Jäger);

**NHMUK**Natural History Museum, London, United Kingdom (Maxwell Barclay, Beulah Garner);

**QM**Queensland Museum, Brisbane, Australia (Geoff Monteith);

**RBINS**Royal Belgian Institut of Natural Sciences, Brussels (Mevr. Martina Peeters);

**ZSM**Zoologische Staatssammlung, Munich (Michael Balke).

Observations of anatomical features were made using a Leica MZ12s stereomicroscope (KW) or Olympus SZX10 (BG). Measurements were made using an ocular reticle except for the holotype of *Adelopomorpha glabra*, for which measurements were estimated using pixel counts from the digital image (image provided by O. Jäger, MTKD). Measurements and calculated ratios for specimens examined are included in the spreadsheet among the Suppl. material 2: Table S1. Suppl. material 2: Table S1 also includes a figure of the measurement points. Measurements and their abbreviations are as follows:

**BL**- body length, sum of HL+PL+EL;

**EL**- elytral length, length of left elytron from the base of the scutellum to the elytral apex;

**EW**- elytral width, width of the widest point of the elytra;

**EyW**- width of head over eyes, width of the head over the eyes at the middle of the eyes’ greatest convexity;

**HL**- length from base of the labrum to estimated based of head;

**HW**- head width, width of the head at the middle of the eye;

**PA**- pronotal anterior width, width between the anterior angles of the pronotum;

**PL**- pronotal length, length along the midline of the pronotum;

**PW**- pronotal width, width of the widest point of pronotum.

Male genitalia and female reproductive tract preparation followed either Will (2020b) or Guéorguiev and Liang (2020). Habitus photos of beetles were taken as image stacks using a modified Microptics XLT digital imaging system that were then aligned and assembled with Helicon Focus version 5.3 (KW), or images were taken using a Canon EOS 600D camera that were then aligned and stacked with Helicon Focus Pro 7.0.2 software (BG). The image files were edited to enhance clarity using standard image editing software.

### Phylogenetic methods

OTU selection (Operation Taxonomic Units). The tribe Oodini has nearly 40 currently recognized genera. An analysis of the entire tribe is beyond the scope of this contribution. As our main purpose is to establish an evidence-based generic placement for the New Caledonian species, we chose exemplar species of three genera to act as outgroup taxa: *Simous* Chaudoir, *Holcocoleus* Chaudoir, and *Evolenes* LeConte. Among Oodini, these genera are plausibly closely related to the putative thryptocerine genera, with *Simous* appearing to be the least apomorphic. The resultant trees were rooted between *Simous* and the remaining OTUs. Species of *Holcocoleus* are included following Erwin’s assumption that this genus may be a possible key to the hypothesized relationships of thryptocerines (Erwin 1974). Both described species of *Holcocoleus*, *H. latus* (LaFerté-Sénectère, 1851) and *H. melanopus* Andrewes, 1936, are subsumed in a single chimeric OTU. This assumes the monophyly of this genus but was necessary due to a limited number of suitable female specimens for dissection and character scoring. *Evolenes exarata* (Dejean, 1831) is a distinctive New World species that has some features common in thryptocerines, such as the structure of the male protarsomeres and the lack of basal pronotal and discal elytral setiferous punctures. A preliminary study by BG of *Lobatodes* suggested a strong similarity to thryptocerine genera and so one of the two species described in the genus, *L. decellei* Basilewsky, 1968 was included.

*Coptocarpus* is by far the largest and most species-rich genus of thryptocerines, and our preliminary study suggested that the New Caledonian species were likely related to some species in the genus. Therefore, Erwin’s species groups are used to structure our sampling to reflect reasonably the diversity of the genus as it is currently circumscribed. In the analysis we included four of the five groups (absent is the *chimbu* group for which we lack specimens) and include an undescribed group-6 species from Mt. Lewis, Queensland.

Both African and New Caledonian *Hoplolenus* are included, as is *Adelopomorpha glabra* and all New Caledonian species and groups designated below. *Hoplolenus insignis* LaFerté-Sénectère, 1851 and *H. obesus* (Murray, 1858) are subsumed in a single chimeric OTU.

The morphological character matrix includes 36 characters from external features, male genitalia, and the female reproductive tract of adult beetles for 24 OTUs. The majority, 26 of the characters, are coded as binary. The 10 multistate characters are treated as unordered. The matrix is largely complete; however, female specimens were not available for five named OTUs and male specimens were not available for two. Additionally, OTUs for the unnamed *glabra* group males and females omit the complementary sex from scoring. In a few cases characters could not be scored due to damaged specimens as noted below. The matrix in both Mesquite and TNT formats (*.nex and *.ss text files, respectively) is included in the Suppl. material 1, 4, 5.

Characters and states used in the analysis:

1. Labral setae number: six (0); four (1).
2. Labral setae position: evenly distributed across width (0); with a central grouping of setae (1).
3. Labral setae arrangement: each in a separate depression (0); medial two lying in a common depression (1); medial four lying in a common depression (2).
4. Clypeal setae: absent (0); with one present each side (1).
5. Supraorbital setae: absent (0); with one present each side (1).
6. Penultimate labial palpomere setation: setose, with one or two setae (0); asetose (1).
7. Elytral microsculpture: isodiametric (0); slightly transverse (1).
8. Elytral striae: all impressed (0); 1–5 partly impressed, 6, 7 entirely impressed (1); none impressed (2).
9. Humeral submarginal carina (Figs 46, 47): absent (0); present (1).
10. Granulation in elytral marginal furrow: continuous for entire length (0); discontinuous, more or less widely absent for a portion of the length (1).
11. Parascutellar punctures: present (0); absent (1).
12. Elytral anterior discal punctures: present (0); absent (1).
13. Elytral posterior discal punctures: present (0); absent (1).
14. Prosternal process shape: rounded (0); rhomboid (1); elongate (2).
15. Mesosternal medial tubercles: none (0); one (1); two (2).
16. Male protarsomeres 1–3 dimorphism: two or more expanded (0); none expanded (1).
17. Male protarsomere 1 shape: transverse, wider than long (0); subelongate (1); elongate, longer than wide (2).
18. Male protarsomeres 3 and 4 basal insertion into 2 and 3, respectively: symmetrically or nearly symmetrically relative to tarsomere width (0); clearly asymmetrically relative to tarsomere width (1).
19. Male protarsomere 1 squamose setae: present overall (0); present on apical 1/2–2/3 of ventral surface (1); present on apical 1/3–1/4 of ventral surface (2); absent (3).
20. Male protarsomere 2 squamose setae: present (0); absent (1).
21. Male protarsomere 3 squamose setae: present (0); absent (1).
22. Male basomesotarsus ventral side: with setae small, thin (as in female) (0); with a series of stout, tooth-like setae (1).
23. Ventral setation on female mesotarsomeres 3 and 4: short and sparse (0); long and dense (1).
24. Ventrites 4 and 5 ambulatory setae: present (0); absent (1).
25. Ventrite 6 ambulatory setae or punctures in males: two (0); none (1).
26. Median lobe apical lamella shape: straight or nearly so (0); slightly curved to right (1); significantly curved to right (2).
27. Sides of apical lamella (blade) of median lobe in dorsal view: straight or convex in both sides (0); concave in one or both sides (1).
28. Basal bulb of medial lobe (dorsal view): rounded dorsally (0); forming edge on dorsal side (1). The only male specimen of A. glabra that was dissected has a damaged basal bulb and could not be scored for this character.
29. Median lobe lamella length: short (0); long (1).
30. Gonocoxite 2 shape: subtriangular (0); elongate (1); falcate (2).
31. Dorsomedial ensiform seta of gonocoxite 2: present (0); absent (1).
32. Lateromedial ensiform seta/e of gonocoxite 2: present (0); absent (1).
33. Spermatheca: elongate, undifferentiated (0); elongate, differentiated seminal canal and receptaculum (1); short, sessile receptaculum (2).
34. Spermatheca diameter: narrow (0); broad (1).
35. Spermatheca form: coiled or twisted (0); straight or nearly straight (1).
36. Spermathecal gland attachment on seminal canal: in apical 1/2 (0); in basal 1/2 (1); on bursa or common oviduct (2).

A parsimony analysis of the morphology matrix for all 24 OTUs was conducted by submitting the matrix to TNT version 1.5 (Goloboff et al. 2008) using implicit enumeration (“IENUM;” command) set in Zephyr version 3.11 package (Maddison and Maddison 2020) within Mesquite version 3.61 (Maddison and Maddison 2019). All files, including the matrix, TNT analysis files, and results files are included in Suppl. material 1, 3–5.

## Results

### Phylogenetic results

**Figure 17.**
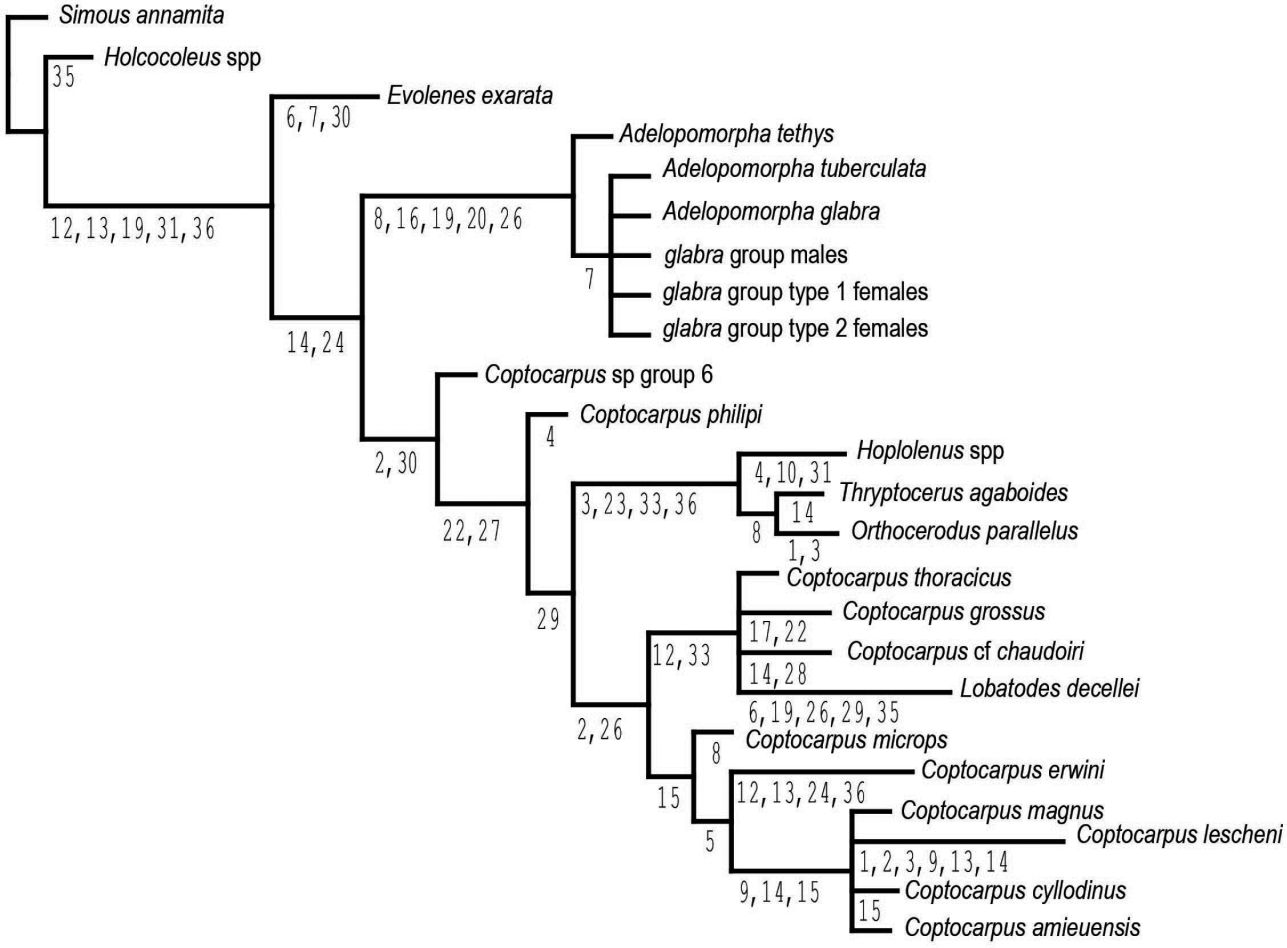
Consensus tree from 30 MPTs resulting from the analysis of adult morphological characters for thryptocerine taxa with character numbers at the points of a state change for those characters that can be unambiguously mapped to all 30 MPTs.

Implicit enumeration examines all possible tree topologies and using this method 30 equally most parsimonious trees of 108 steps, c.i. of 0.46, and r.i. of 0.65 were found (length calculated using TNT). All MPTs (most parsimonious trees) are including in Suppl. materials 1–5. The strict consensus of those 30 trees is largely resolved (Fig. 17). Thryptocerina, and *Adelopomorpha* are each monophyletic. *Coptocarpus* as conceived previously is not monophyletic. New Caledonian species of *Coptocarpus* do form a clade, but the Australian species are rendered paraphyletic by a clade of *Hoplolenus* (excluding “*H. cyllodinus*”), *Thryptocerus agaboides* (Fairmaire, 1868), and *Orthocerodus parallelus* Jeannel, 1949. Additionally, *Lobatodes decellei* groups with several species of *Coptocarpus*. The status of *Coptocarpus* and other thryptocerine genera in light of these results is discussed below. All New Caledonian species are placed in either *Adelopomorpha* or *Coptocarpus* as circumscribed herein.

### Systematics

#### Check list of New Caledonian Oodini

*Coptocarpus*

*microps* group

*C. microps* sp. nov.

*erwini* group

*C. erwini* sp. nov.

*cyllodinus* group

*C. cyllodinus* (Fauvel, 1882), comb. nov.

*C. amieuensis* sp. nov.

*C. magnus* sp. nov.

*lescheni* group

*C. lescheni* sp. nov.

*Adelopomorpha*

*tethys* group

*A. tethys* sp. nov.

*glabra* group

*A. tuberculata* sp. nov.

*A. glabra* Heller, 1916

unnamed *glabra* group males

unnamed *glabra* group female type 1

unnamed *glabra* group female type 2

#### Oodini LaFerté-Sénectère,1851

##### Thryptocerina

Taxon classificationAnimaliaColeopteraCarabidae

Jeannel, 1949

1078FD21-F43E-52E2-B514-DE8621EA51CE

Thryptocerini Jeannel, 1949: 775, 829, 841. Type genus: Thryptocerus Chaudoir, 1878: 74.

###### Diagnosis.

Male protarsomeres 2–4 eccentrically attached to the preceding tarsomere (with basal axis of the former affixed on lateral 1/2–1/3 of the latter); squamose setae only on apical 1/3 or 1/2 of ventral surface of male protarsomere 1 (except in *Adelopomorpha* species that have narrow, symmetrically attached male protarsomeres 2–4 lacking any such setae, a derived loss of the two characteristics). Pronotum without setiferous punctures near the hind angles. Parascutellar seta present, puncture to accommodate it rather large, foveate. Abdominal ventrites 1–5 without ambulatory setae (present as a reversal on ventrites 3–5 only in *Coptocarpus erwini*).

**Figures 18–21.**
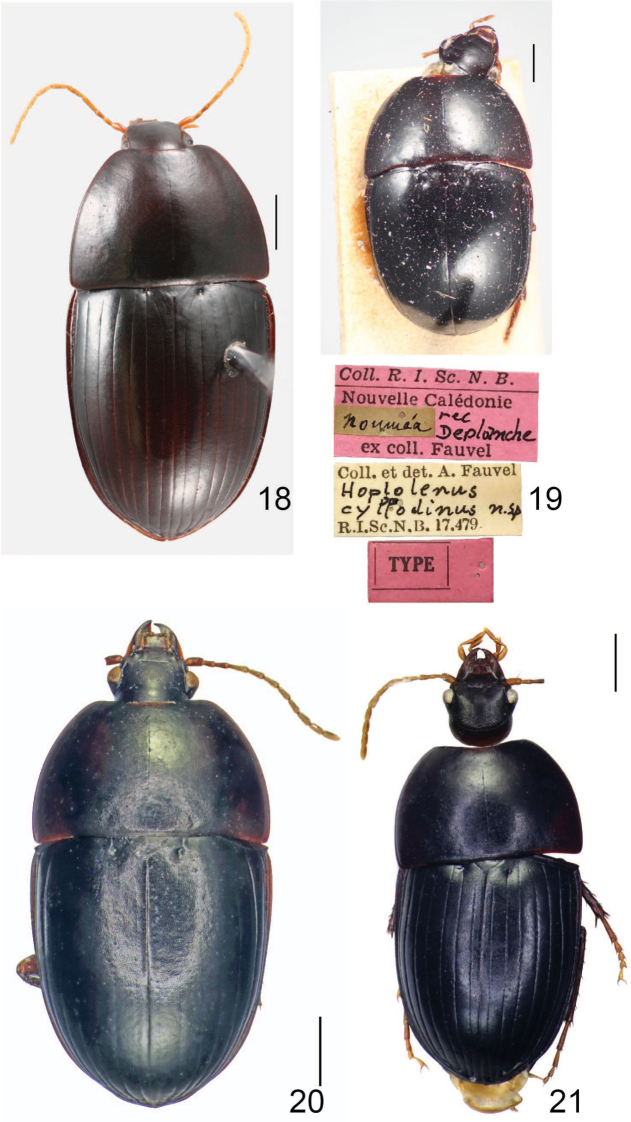
Dorsal habitus of **18***C. erwini* sp. nov. **19** holotype of *Coptocarpus cyllodinus*, with labels **20***C. amieuensis* sp. nov. **21***C. lescheni* sp. nov. Scale bars: 1.0 mm.

Recognition among the Carabidae of New Caledonia. All oodines are distinguished by having the elytral intervals 7 and 8 fused posteriorly, forming a ridge over a deeply impressed stria 8 that extends to the suture, and having the metepisternum laterally coadunate with the epipleuron. All are broadly oval, very convex (Figs 18–21, 41, 42), black with the head and legs often reddish brown or rufous, have the flight wings reduced, and the metasternum and metepisternum very short. The general form is similar to some smaller species of *Cerabilia* Laporte (Abacetini) that are found in rainforest leaf litter with oodines in New Caledonia. *Cerabilia* species are readily separated by the presence of two supraorbital setae, presence of marginal pronotal setae, the lack of an elytral plica, and the male protarsomeres ventrally biseriate squamulose, compared to oodine species with one or no supraorbital and no pronotal setae, a large elytral plica, and the male protarsomere squamulose with the setae forming broad pads or being entirely unmodified.

##### Coptocarpus

Taxon classificationAnimaliaColeopteraCarabidae

Chaudoir, 1857

347DDADC-F094-5D06-9FE2-69F1B7DA5EAE

###### Type species.

*Oodes australus* Dejean, 1831: 671, by original designation.

New Caledonian *Coptocarpus* species have the characteristics of the genus as described by Erwin (1974), with the additions noted below. The New Caledonian species are small (BL 6.5–9.6 mm) compared to Australian species (7.4–15.0 mm); antennae pubescent from antennomere 4 or 2; clypeus with or without setiferous puncture each side; supraorbital pair of setae of head (one seta each side) present or absent; elytral marginal furrow with or without humeral submarginal carina, with or without continuous granulation; elytral discal punctures in interval 3 present or absent; male protarsomeres 1–3 asymmetrically dilated, 2 and 3 fully clothed beneath with squamose setae that are transversely arranged or protarsomeres not expanded and not squamulose ventrally; male basomesotarsus modified or not; abdominal ventrites 1–5 with or without a pair of submarginal ambulatory setae; spermatheca short and straight, or rather long, coiled distally, with spermathecal gland attached to basal or medial 1/3 of seminal canal.

###### Description of the New Caledonian Clade of *Coptocarpus*.

***Habitus***. Medium sized, BL: 6.50–9.60 mm, BW 3.00–4.40 mm, ovate, convex body. ***Chaetotaxy***. Labrum with four or six setae, lateral two setae longer than medial two or four. Supraorbital setae one each side or absent. Anterior seta of stipes present. Ventral seta of antennomere 2 present and long. Pair of long setae on apical margin of ligula. Penultimate labial palpomere glabrous. Mentum with two long, paramedial setae. Submentum with two long, posterolateral setae. Pronotum without setiferous punctures. Elytron without discal setiferous punctures in interval 3, or rarely posterior punctures present, but very small and lacking a seta (*C. lescheni* and *C. erwini*); parascutellar seta present, puncture very large, foveate. Mesocoxa with posteromedial and lateral setae; mesotrochanter with seta. Hind femur without posterior setae. Abdominal ventrites 3–5 with (*C. erwini*) or without (all other species) ambulatory setae; last ventrite with two setae in male and four setae in female. Gonocoxite 2 with a large dorsomedial seta, without lateromedial ensiform setae. ***Head*.** Labrum rectangular, flat or slightly concave medially, shorter than clypeus. Mandible narrow, long, strongly to moderately curved at tip with sides convex or nearly straight. Apical maxillary palpomere slightly pointed at apex, as long as penultimate one. Mentum tooth triangular, with apex pointed. Gula smooth, somewhat convex. ***Thorax*.** Pronotum with sides evenly, very shallowly rounded from anterior angles to posterior ones; maximal width in posterior 1/5. Disc slightly to moderately convex, without laterobasal impressions; basal margin, shallowly sinuate, without bead, posterior angles slightly obtuse or ~ right angled, widely rounded; lateral bead distinct, complete, ended at posterior angles. Prosternum with median longitudinal sulcus distinct, shallowly impressed (most species) or indistinct (*C. cyllodinus*). Mesosternum deeply concave, with or without medial tubercle (situated just posterior to mesosternal collar). ***Elytra*.** Disc convex. Basal margin distinct laterally, ended medially near level of striae 2 or 3. Humeral submarginal carina present or absent. Interval 9 transformed into marginal furrow; granulation in marginal furrow continuous (*C. lescheni*) or discontinuous (most species). ***Legs*.** Metacoxal basal sulcus short, ended at midpoint. Submedial assemblage of mesotibial setae mostly of two or three setae (most species), rarely three or four (*C. lescheni*). Basomesotarsus from above glabrous (most species) or with few short, scattered setae (*C. lescheni*); male basomesotarsus more or less flattened (most species) or constricted basally and flattened apically (*C. erwini*), ventrally with some very short, stout setae in apical 2/3; female basomesotarsus not flattened, ventrally with finer setae; both sexes with mesotarsomere 2 ventrally with shorter and less dense setae than those on mesotarsomeres 3 and 4, and mesotarsomere 5 glabrous ventrally. ***Female genitalia*.** Bursa copulatrix relatively large in relation to spermatheca. Spermatheca elongate and narrow, coiled or twisted in distal 1/2, differentiated into seminal canal and receptaculum or undifferentiated; spermathecal gland connected near basal 1/6 or medial 1/3 of spermatheca, spermathecal canal forming short atrium, separated from gland. Common oviduct long, connected to bursa. ***Male genitalia*.** Median lobe of aedeagus in lateral view long, curved ventrally; lobe in dorsal view with apical lamella long, significantly bent to right, with right side concave and left side straight or slightly convex; ostium long, reaching basal bulb; basal bulb short; sclerotized portion of endophallus with one or three sclerites.

##### *microps* group

**Notes.** This group includes a single species that lacks impressed elytral striae and the humeral submarginal carina, both apparent plesiomorphic states. The reduced eye is distinctly smaller than in other New Caledonian congeners and appears to be the only apomorphy for the group.

##### Coptocarpus microps sp. nov.

Taxon classificationAnimaliaColeopteraCarabidae

A8615003-4D66-59F3-A279-50444B769510

http://zoobank.org/F40E107F-8537-4EC8-B245-6DBE22E18316

**Figures 22–31.**
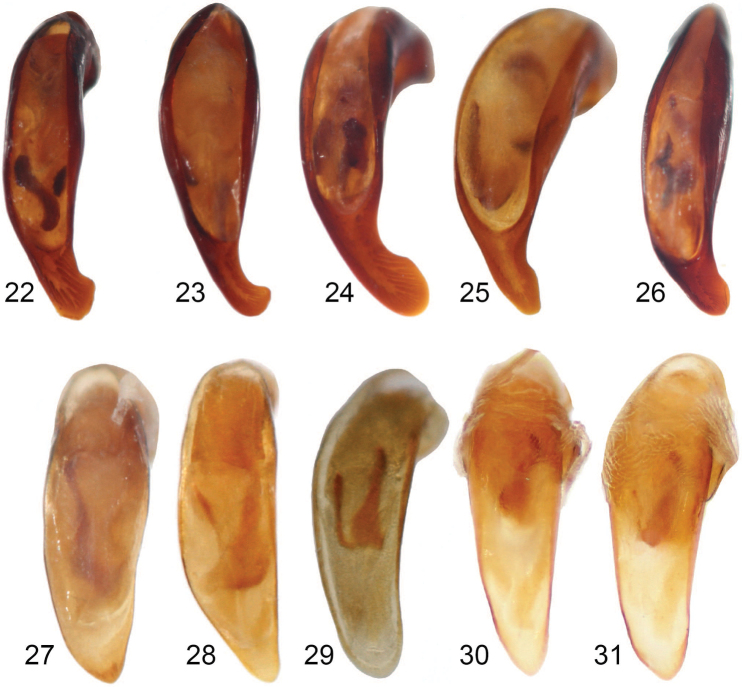
Aedeagus, dorsal view of **22***Coptocarpus microps* sp. nov. **23***C. erwini* sp. nov. **24***C. cyllodinus* (Fauvel) **25***C. amieuensis* sp. nov. **26***C. magnus* sp. nov. **27***Adelopomorpha tethys* sp. nov. **28***A. tuberculata* sp. nov. **29***A. glabra* Heller **30***glabra* group male from Mt. Koghis **31***glabra* group male from Gelima.

**Figures 32–40.**
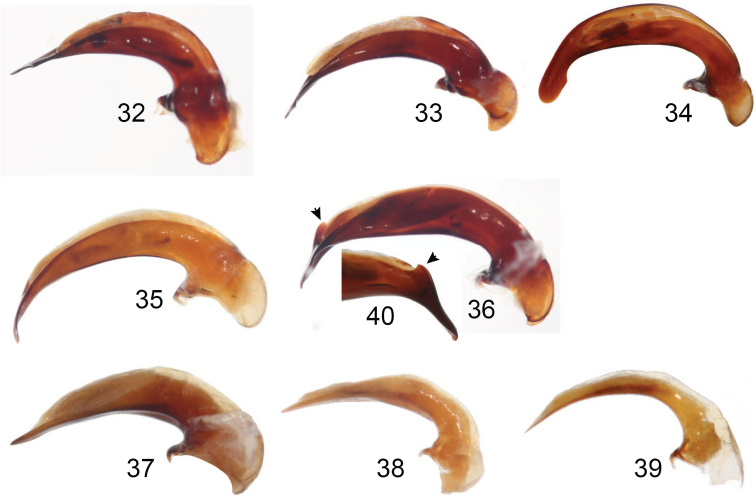
Aedeagus, left lateral view of **32***Coptocarpus microps* sp. nov. **33***C. erwini* sp. nov. **34***C. cyllodinus* (Fauvel) **35***C. amieuensis* sp. nov. **36***C. magnus* sp. nov. **37***Adelopomorpha tethys* sp. nov. **38***A. tuberculata* sp. nov. **39***A. glabra* Heller **40** right lateral view of tip with arrow indicating the dorsal tooth of *C. magnus*.

Figs 22 , 32 , 49 , 53

###### Material examined.

***Holotype***: New Caledonia • ♂ “NEW CALEDONIA 21°10'Sx165°18'E, Mt. Aoupinié, May–Oct 1992, R. Raven & E. Guilbert, rainforest, pitfalls” Holotype pinned, with genitalia in a separate microvial. Source collection QM, deposited MNHN. Type locality as given on label.

###### Diagnosis.

Similar to *Adelopomorpha tethys* in size and the lack of impressed striae 1–5, but with very small, flat eyes and male with protarsomeres 1–3 expanded. Both *A. glabra* and *A. tuberculata* also lack impressed striae, but are much smaller, and in *C. microps* the male has a pair of setae on ventrite 6 that is lacking in those species. The aedeagus is decisively different from all other species.

###### Description.

***Habitus*.** Small sized, BL: 6.80 mm and BW: 3.00 mm, ovate, convex body. ***Color and luster*.** Head, prothorax, and legs deep reddish brown. Pronotal disc and apical portion of prosternal process darker, nearly black. Elytra and ventrites mostly black, paler reddish black on elytral epipleura and near humeri, and medially on ventrite 6; antennae and palpi reddish brown. Integument moderately dull, without spectral iridescence. ***Microsculpture and punctation*.** Dorsal surface of head and pronotum with prominent isodiametric meshes; elytra with somewhat irregular and slightly less prominent isodiametric meshes throughout, more irregular or very slightly stretched in apical 1/3; ventral surface with scarcely-visible sculpticells or mostly sculpticells not apparent. Head lacking macropunctation or wrinkles on vertex; head, pronotum and elytra with scattered micropunctation, only on elytra do micropunctures and sculpticells form some irregular rosettes (sensu Spence 1983); abdominal ventrites 1–3 at sides with several large punctures (more irregular in ventrite 3), ventrites 4 and 5 at sides with few coarse wrinkles, and ventrite 6 nearly smooth. ***Chaetotaxy*.** Labrum with six setae of nearly equal length each in its own socket, lateral setae slightly larger than medial four. Clypeal setae present near apicolateral corners. Single supraorbital seta present over each eye. Elytron without discal setiferous punctures. Abdominal ventrites 1–5 without ambulatory setae. ***Head*.** Approximately 1/3 as wide as pronotum (Suppl. material 2: Table S1). Eye very small, slightly protruded, EyW/HW: 1.17. Labrum anterior margin straight. Frontoclypeal sutures not evident, marked by a very broad, shallow depression. Antenna moderately long, with last segment exceeding pronotal base and pubescence starting from antennomere 4. Last labial palpomere slightly swollen, blunt at apex, slightly longer than penultimate one. Mentum without paramedial border. ***Thorax*.** Pronotum just slightly less than 1 2/3 wider than long (PW/PL: 1.61); width at apex 2 1/2 × less than at widest point (PW/PA: 2.52). Disc with middle line fine, very shallow, and ended well before apical and basal margins, without apical transverse impression; anterior angles not prominent, scarcely convex at tips, anterior margin shallowly concave, submarginal sulcus present near angles, lacking in middle 1/3. Prosternal process rounded, bordered throughout. Mesosternum with single, low medial tubercle. Metepisternum 1 1/3 × wider than long, with lateral margin nearly straight, coadunation with epipleuron along entire length. ***Elytra*.** Approximately 1/3 longer than wide (EL/EW: 1.27). Basal margin forming extremely minute, blunt denticle at shoulder, ended medially at level of parascutellar punctures, joined to parascutellar puncture fovea by a short striole. Humeral submarginal carina absent. Apical sinuation evident but shallow. Disc with no elytral striae impressed, those marked by very shallow, fine punctures; parascutellar striole absent. Granulation in marginal furrow discontinuous, interrupted broadly at midlength. ***Legs*.** Mesotibia notably dilated apically. Basomesotarsus and basometatarsus glabrous dorsally; basomesotarsus broadly flattened, with seven or eight very short, stout setae ventrally. ***Female genitalia*.** Female specimens unknown. ***Male genitalia*.** Median lobe of aedeagus in lateral view long, almost straight, with thin apex (Fig. 32); lobe in dorsal view, with apical lamella well bent to right, with right side concave and left side straight (Fig. 22); basal bulb rounded dorsally; sclerotized portion of endophallus visible in repose, with three sclerites, two near base of endophallus and one near apex.

###### Etymology.

The specific epithet *microps* is Latin for small eyes and draws attention to the extraordinary small eyes in these beetles (Fig. 49). It is treated as an adjective in the nominative singular.

##### *erwini* group

**Notes.** The single included species has a very unique combination of character states among all thryptocerines. It has plesiomorphic states such as the absence of the submarginal humeral carina (Fig. 46) found in more derived members of the New Caledonian *Coptocarpus* clade; abdominal ventrites 3–5 with ambulatory setae, which is an apparent reversal from the absence of these setae in all other thryptocerines; and spermathecal gland attached near apical 1/2 of spermatheca, an apomorphy for *Coptocarpus* and Thryptocerina and likely a convergent state shared by the distantly related *Holcocoleus*. It is also apomorphic and unique in having an elongate, somewhat flattened head, emarginate labrum, and somewhat straight, elongate mandibles with a very small terebral tooth.

##### Coptocarpus erwini sp. nov.

Taxon classificationAnimaliaColeopteraCarabidae

BA3D575E-698E-5A26-9879-8036B5AA7E67

http://zoobank.org/0C0F90B7-37C7-48DF-B2C6-4F5E55325E1C

Figs 11 , 18 , 23 , 33 , 46 , 53

###### Material examined.

***Holotype***: New Caledonia • ♂ “NEW CALEDONIA 11138 21°53'S x166°24'E,1400m Mt. Humboldt, moss forest, 6–7 Nov2002, Monteith & Burwell, pyreth, trees & logs” Holotype pinned, with genitalia in a separate microvial, abdominal ventrites and leg glued to point. Source collection QM, deposited MNHN. Type locality as given on label.

***Paratypes***: New Caledonia • ♀; same data as holotype [QM] • ♀; “NEW-CALEDONIE Nouméa Mt. Koghi // 23.II.1997 leg. Dr. J. BALOGH” [ZSM] • ♂; “NEW CALEDONIA 11131 21°53'Sx166°25'E, 1350 m Mt Humboldt refuge, Night collecting, 5–8 Nov 2002, Burwell, Monteith & Wright” [QM].

###### Diagnosis.

*Coptocarpus erwini*, *C. lescheni*, and *C. magnus* all have completely impressed elytral striae. Of these three only *C. erwini* lacks both the elytral submarginal carina (Fig. 46) and the apicolateral setae of the clypeus (present in *C. magnus*). In *C. erwini* the antennae are pubescent from antennomere 4 (pubescent from antennomere 2 in *C. lescheni*).

###### Description.

***Habitus*.** Medium sized, BL: 8.55 mm and BW: 3.65 mm (range, BL: 8.35–8.80 mm, BW 3.55–3.65 mm) ovate, convex body. ***Color and luster*.** Head and pronotum reddish black, elytra black to dark reddish black; ventral surface of body reddish brown to black; legs deep reddish brown, tarsomeres paler brown. Abdominal ventrites reddish black medially and apical edge of ventrite 6; antennae and palpi brown. Integument moderately dull, without spectral iridescence except irregularly ventrally. ***Microsculpture and punctation*.** Dorsal surface of head, pronotum, and elytra with prominent isodiametric meshes; ventral surface with scarcely-visible sculpticells or mostly sculpticells not apparent except on ventrite 6 and apically on prosternum. Head lacking macropunctation or wrinkles on vertex; head, pronotum and elytra with scattered micropunctation; abdominal ventrites 1–3 laterally with coarse punctures, becoming finer and less extensive on 3; ventrites 3–5 very shallowly wrinkled laterally, 4 and 5 smooth medially, and 6 smooth throughout. ***Chaetotaxy*.** Labrum with six setae, each in its own socket, lateral setae longer than medial four, which are set more closely to each other than to the lateral setae. Clypeal setae absent. Single supraorbital seta absent. Elytron with two discal setiferous punctures in interval 3 (some× represented by only a puncture). Abdominal ventrites 3–5 with ambulatory setae. ***Head*.** Approximately 1/3 as wide as pronotum (Suppl. material 2: Table S1). Eye size moderate, eye somewhat protruded, EyW/HW: 1.30 (range 1.24–1.30). Labrum with anterior margin deeply emarginate. Frontoclypeal sutures not evident. Antenna moderate length, with last segment nearly reaching pronotal base and pubescence starting from antennomere 4. Last labial palpomere elongate, blunt at apex, length ~ equal to penultimate one. Mentum tooth without paramedial border. ***Thorax*.** Pronotum elongate, 1 1/2 × wider than long (PW/PL: 1.54, (range, 1.48–1.54)); width at apex 2 2/3 × less than at widest point (PW/PA: 2.37 (range, 2.22–2.41)). Disc with midline fine, well impressed, and ended well before apical and basal margins, without apical transverse impression; anterior angles scarcely convex, anterior margin shallowly concave, submarginal sulcus present near angles, lacking in middle 3/5. Prosternal process acuminate, bordered throughout. Mesosternum with single, low medial tubercle. Metepisternum 1 1/3 × wider than long, with lateral margin produced posteriorly, coadunation with epipleuron along entire length. ***Elytra*.** Approximately 1/3 longer than wide (EL/EW: 1.27 (range 1.25–1.30)). Basal margin forming prominent, sharp tooth at shoulder, ended medially at level of stria 3. Humeral submarginal carina absent. Elytral apical sinuation not evident. Disc with all striae impressed; parascutellar striole absent or marked by a small, shallow remnant. Elytral intervals flat; granulation in marginal furrow discontinuous, interrupted broadly at midlength. ***Legs*.** Mesotibia slightly curved and moderately dilated apically. Basomesotarsus and basometatarsus glabrous dorsally; basomesotarsus in male constricted basally, apically flattened, with 11 or 12 short, stout setae ventrally and many smaller setae scattered on lateral portions. In female, basomesotarsus cylindrical and with scattered, fine dorsal setae and rows of moderately thicker ventral setae. ***Female genitalia*.** Gonocoxite 2 elongate. Spermatheca narrow and long, coiled in distal 1/2, undifferentiated; spermathecal gland connected near medial 1/3 of spermatheca. ***Male genitalia*.** Median lobe of aedeagus in lateral view long, almost straight, with thin apex slightly flexed ventrally on the left lateral edge (Fig. 33); lobe in dorsal view with apical lamella well bent to right, with right side deeply concave and left side straight (Fig. 23); basal bulb forming edge dorsally with evident crest; sclerotized portion of endophallus visible in repose with one evident sclerite.

###### Etymology.

The specific epithet *erwini* is treated as a noun in the genitive case and is in honor of Terry Erwin and his amazing, life-long contribution to carabidology.

**Figures 41, 42.**
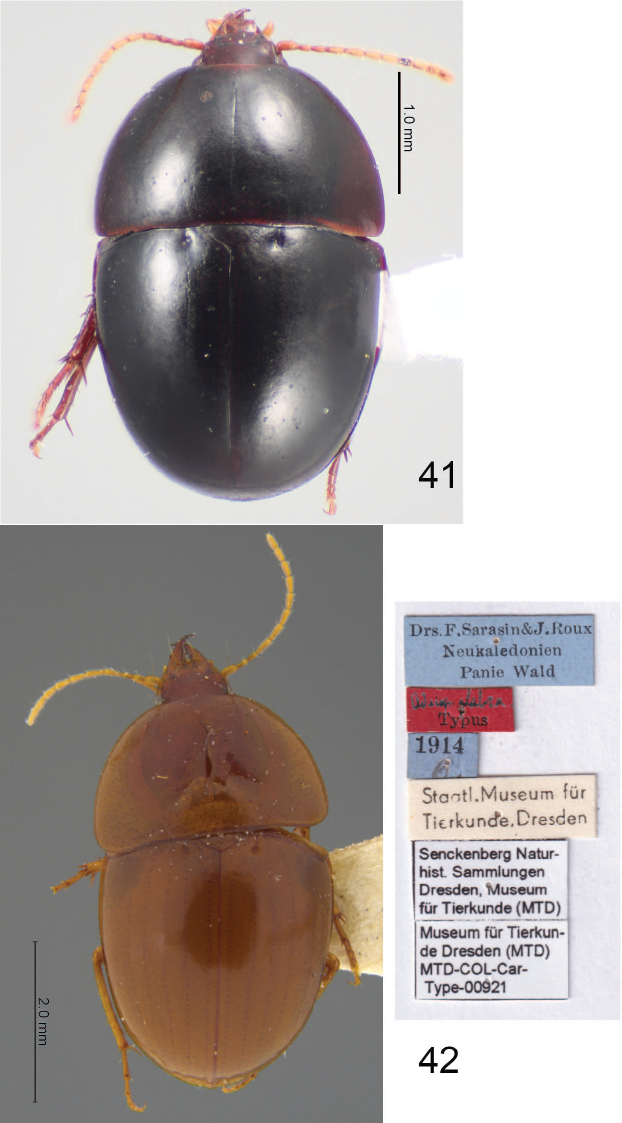
Dorsal habitus of **41***Adelopomorpha tuberculata***42** holotype of *A. glabra* Heller, with labels. Images of *A. glabra* by O. Jäger, reproduced with permission from MTKD.

**Figures 43, 44.**
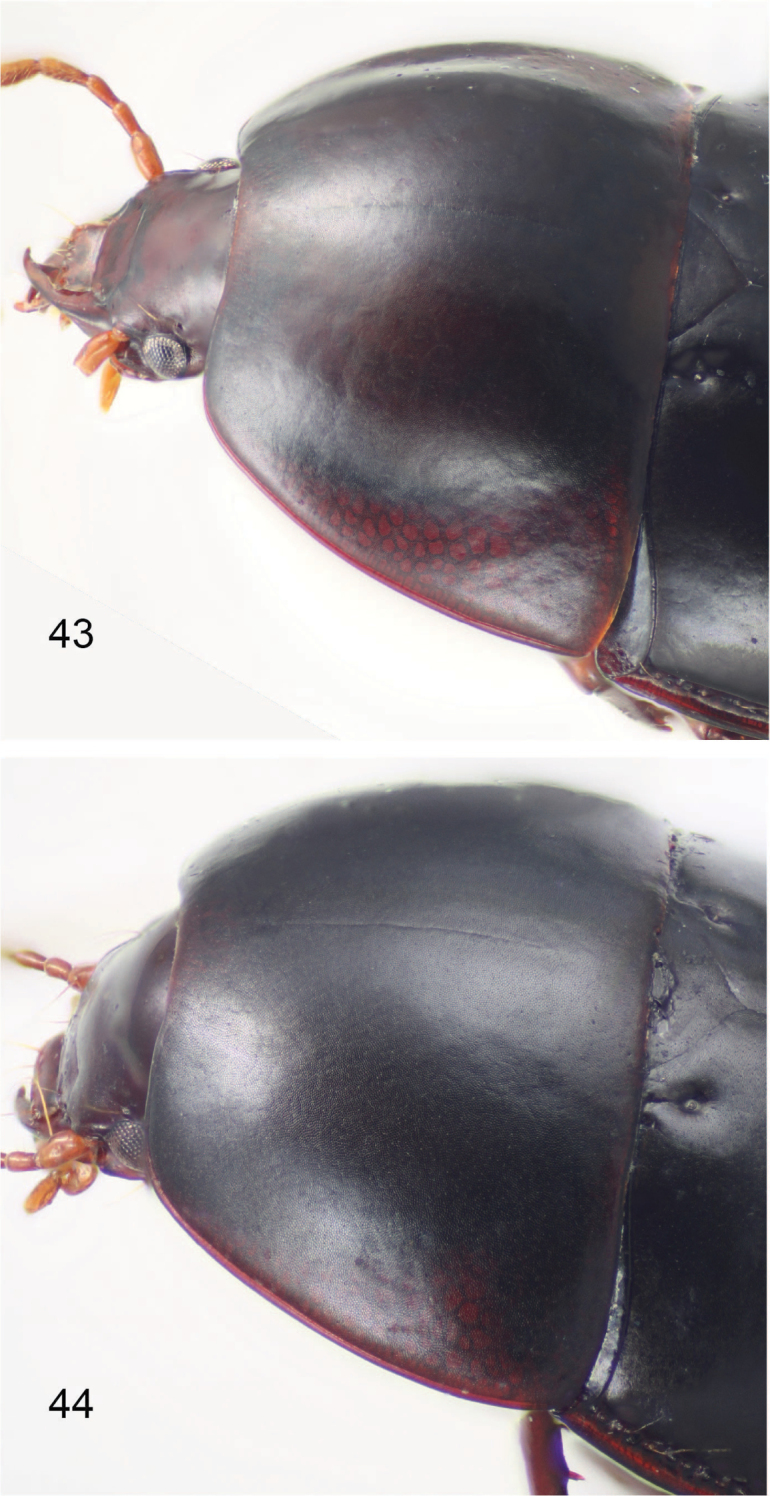
Forebody, left dorsolateral view of **43***Adelopomorpha glabra* Heller **44***glabra* group male from Gelima.

##### *cyllodinus* group

**Notes.** Includes species with antennae pubescent from antennomere 4, prosternal process shape rhomboid, and humeral submarginal carina present (Fig. 47). The presence of the humeral submarginal carina is a unique and unreversed synapomorphy for this group and the *lescheni* group.

##### Coptocarpus cyllodinus

Taxon classificationAnimaliaColeopteraCarabidae

(Fauvel, 1882) comb. nov.

2F6758F0-659B-5A2F-B018-4BB2FD61DCAE

Figs 12 , 19 , 24 , 34 , 47 , 48 , 50 , 52

Hoplolenus cyllodinus Fauvel, 1882: 266.

###### Type locality.

Nouméa.

###### Material examined.

***Holotype***: New Caledonia • ♂, card-mounted specimen, original label with “Nouméa” glued to pink label “Coll. R.I.Sc.N.B. Nouvelle Calédonie rec Deplanche ex coll. Fauvel”// “TYPE” // “Coll.et det. A. Fauvel *Hoplolenus cyllodinus* n. sp. R.I.Sc.N.B. 17.479” [RBINS].

###### Other material.

New Caledonia • ♂; Mt. Koghis; 22°11'S, 166°31'E; track entrance; 500 m; 6.v.2006; berleseate; G.B.Monteith; [QM] • ♀; Mt. Koghis; 22°11'S, 166°00'E; 750 m; 29.xi.2000; berleseate, sieved litter, rainforest; G.B. Monteith; [QM] • ♂; 22°10'28"S, 166°30'48"E, 700 m, 12.iii.2007; K. Will; EMEC1075689; [EMEC] • ♂; same data as for preceding; headlamp search; EMEC1075690; [EMEC] • ♂; same data as for preceding; under rocks/logs; EMEC1075691; [EMEC] • ♂; Nouméa Mt. Kogni; 12–13.II.1977; leg. Dr. J. BALOGH; [NHMUK] • ♀; “Kuakui” [Mt. Kouakoué]; P.D. Montague; B. M. 1946-210; [NHMUK] • ♂; Yahoué; “février”; [RBINS, Fauvel collection, but not a syntype] • ♂; Riviére Bleue Park: 22°09'00"S, 166°41'12"E, 330 m, Houp Géant trail, 13.iii.2007, headlamp search, K. Will; EMEC1075688; [EMEC] • ♀; same data as for preceding; [EMCE] • ♂; Mt. Dzumac; 22°03'S, 166°28'E; Mt. Dzumac Road; 700 m; 1.xii.2000; berleseate, sieved litter, rainforest; G.B. Monteith; [QM] • ♂; same data as for preceding; [ZSM] • ♀; Col de Yate; 22°10'S, 166°55'E; 2.xi.2004; hand collecting, CJ Burwell; [QM] • ♀; Pic du Pin; 22°14'S, 166°50'E; 280 m; 25–26.xi.2004; day hand collecting, rainforest; QM party; [QM].

**Figure 45.**
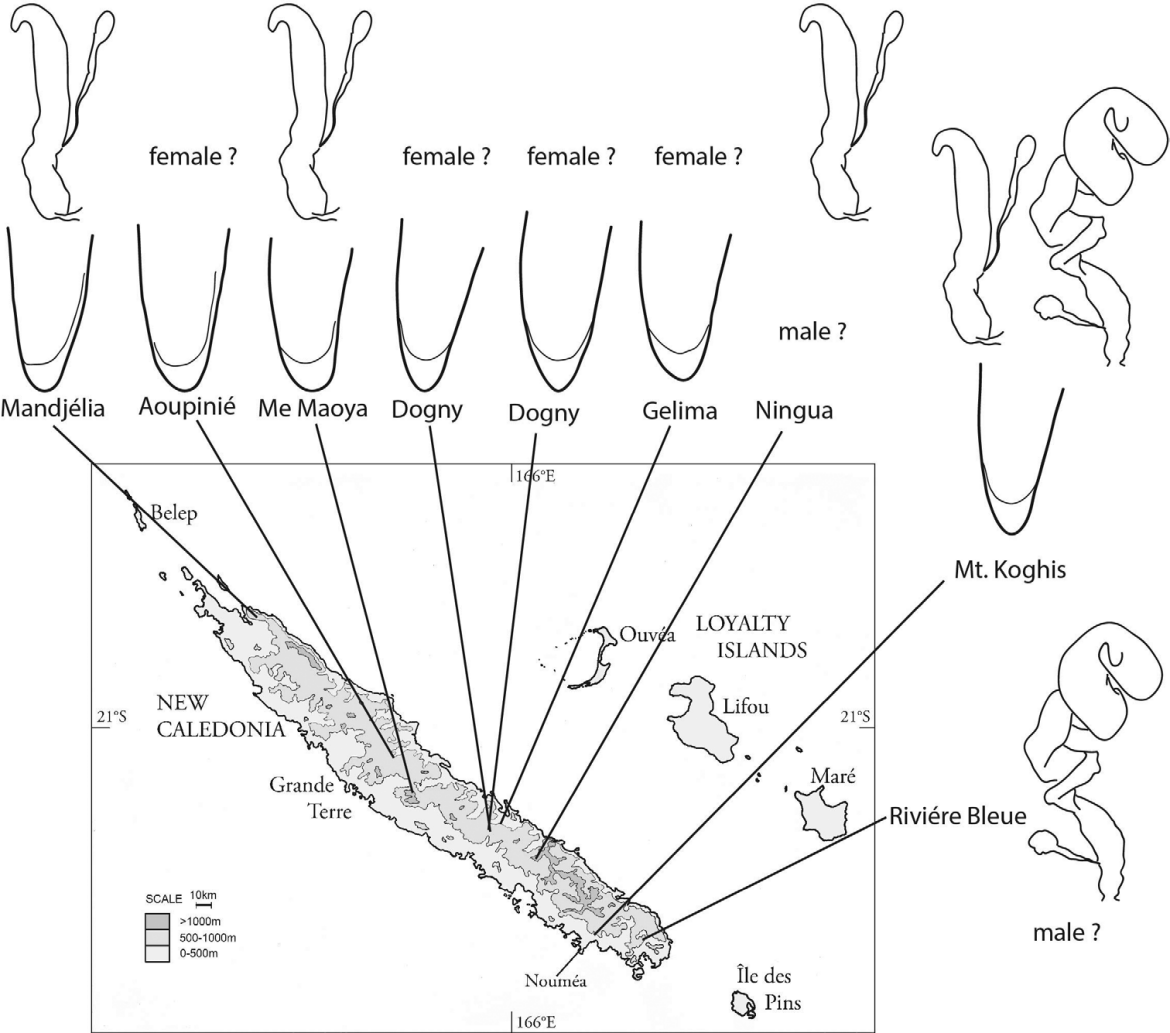
A diagram of the variation in aedeagus tip form and spermatheca types included in the *glabra* species group. Outlines of the aedeagi are based on dissected males from the listed locality. Spermatheca and gland outlines are somewhat stylized, showing generalized type as in characters 34–37.

**Figures 46, 47.**
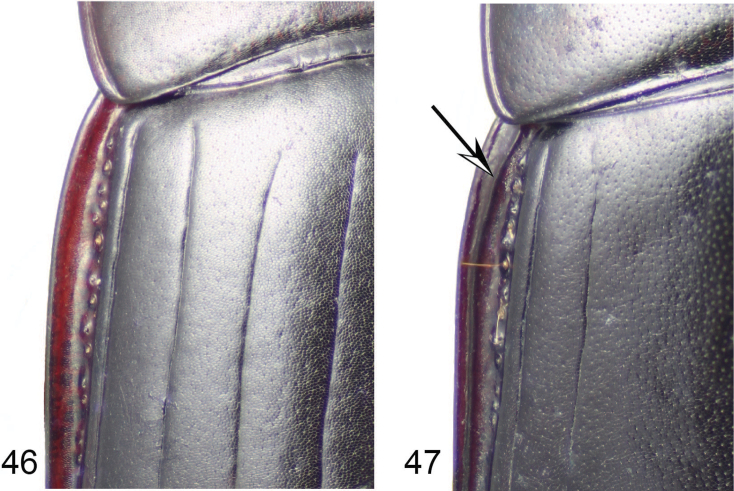
Humeral region of left elytron of **46***Coptocarpus erwini* sp. nov. **47***C. cyllodinus* (Fauvel). Arrow indicates the submarginal carina.

###### Diagnosis.

**Figure 48.**
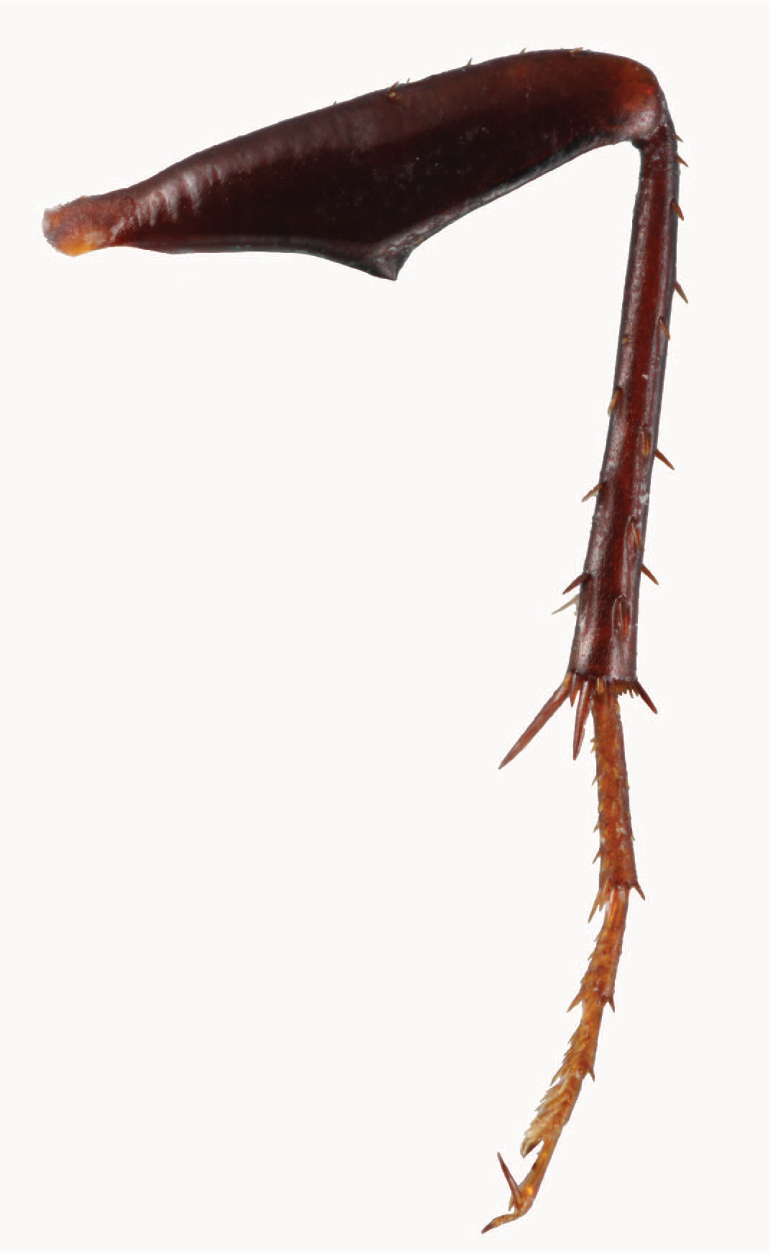
Left hind leg, anterior view of *Coptocarpus cyllodinus* (Fauvel) male.

A very distinctive, large species for the New Caledonian fauna with a deep notch below the eye that may receive the antenna (Fig. 50); eye and orbit very prominent, almost shelf-like; male metafemur with large, ventral tooth (Fig. 48), clypeal setae absent, elytral stria 6 and 7 impressed throughout, 5 only impressed in apical 1/2, 1–4 not evident or only shallowly impressed near the apex. Aedeagus with distinct, broad apical blade (Fig. 24).

###### Redescription.

***Habitus*.** Large sized for New Caledonia oodines (BL: 7.35–8.45 mm, BW: 3.60–4.40 mm), with elliptic, moderately convex body (Fig. 19). ***Color and luster*.** Head dark reddish black to black; clypeus and mandibles reddish brown; palpi, antennae and tarsi yellow to yellow brown; pronotum, elytra and ventral surface of body black; legs (excl. tarsi) deep reddish brown to reddish black. Integument moderately shiny, mostly without spectral iridescence, excluding proepisterna and abdominal ventrites on sides where it is present. ***Microsculpture and punctation*.** Dorsal surface of head (excluding mandibles) and pronotum, with isodiametric meshes well impressed and visible; elytra with isodiametric meshes weakly visible; prosternum with scarcely-visible isodiametric sculpticells, ventrites 4–6 medially with scarcely-visible slightly transverse meshes, rest of ventral surface with sculpticells mostly not evident. Dorsal surface lacking macropunctation; head, pronotum and elytra with micropunctation, smaller on head and most of pronotum, larger on basal 1/4 of pronotum and elytra; micropunctation regular on metacoxae, very scattered on last abdominal ventrites, absent on rest of ventral surface, abdominal ventrites 1–3 macropunctate on sides, 2–5 finely coarsely wrinkled on sides. ***Chaetotaxy*.** Labrum with six setae, each in its own socket, four lateral setae larger than medial two. Clypeal setae absent. Supraorbital setae absent. Elytron without discal setiferous punctures. Abdominal ventrites 1–5 without ambulatory setae. ***Head*.** Approximately 2 1/2 × as wide as pronotum (Suppl. material 2: Table S1). Eye size large, eye prominently protruded, EyW/HW: 1.38–1.48. Labrum anterior margin somewhat concave. Frontoclypeal suture evident, but slightly impressed. Antenna relatively short, with pubescence starting from segment 4. Last labial palpomere swollen, blunt at apex, longer and larger than penultimate one. Mentum tooth with paramedial border distinct. ***Thorax*.** Pronotum ~ 1 2/3 wider than long (PW/PL: 1.64–1.85); width at apex 2–2 2/3 × less than at widest point (PW/PA: 1.95–2.38). Disc with midline fine, shallowly impressed, and ended well before apical and basal margins, without apical transverse impression; anterior angles weakly projected, rounded at tips, anterior margin concave, submarginal sulcus laterally present, lacking in medial 3/10. Prosternal process rhomboid, elongate, broadly pointed at apex, bordered at apex, not bordered at sides. Mesosternum anteriorly without medial tubercle. Metepisternum ~ 1 1/2 × wider than long, with lateral margin convex, coadunation with epipleuron long, located medially and posteriorly; suture between metepisternum and metepimeron barely distinct. ***Elytra*.** Broad, slightly longer than wide (EL/EW: 1.05–1.17). Basal margin forming small, sharp tooth at shoulder, medially nearing parascutellar puncture to which it is joined by impressed basal section of stria 2. Humeral submarginal carina present. Apical sinuation shallow, barely distinct. Elytral striae 6 and 7 entirely impressed, 5 impressed only at posterior 1/3, 3 and 4 only at apical 1/7 or 1/6, anterior continuations of striae 3–5, whole of striae 1 and 2 and parascutellar striole not impressed. Granulation in marginal furrow discontinuous, interrupted briefly along second elytral quarter. ***Legs*.** Mesotibia evenly dilated apically. Male protarsomeres 1–3 expanded, 1 subelongate, nearly as long as wide, 2 and 3 wider than long; ventral adhesive setae present only at apical 1/3–1/4 surface of protarsomere 1 and entire surfaces of 2 and 3. Male basomesotarsus slightly flattened, with apical 2/3 provided with some short, stout, tooth-like setae ventrally; female basomesotarsus with finer setae ventrally. ***Female genitalia*.** Gonocoxite 2 subtriangular. Spermatheca narrow and long, nearly straight in basal 1/2, coiled in distal 1/2, undifferentiated; spermathecal gland connected near basal 1/6. ***Male genitalia*.** Median lobe of aedeagus in lateral view long, considerably bent ventrally, tapering distally, slightly broadened at apex (Fig. 34), with relatively large basal bulb; lobe in dorsal view with apical lamella strongly bent to right, with right side quite concave and left side slightly and evenly convex (Fig. 24); basal bulb forming edge dorsally with evident crest; sclerotized portion of endophallus visible in repose with three sclerites.

###### Notes.

Fauvel stated that this species has only four setae on the anterior edge of the labrum. However, the type specimen has a slightly malformed labrum with three setae on the right and two on the left. The median seta on the right is minute.

##### Coptocarpus amieuensis sp. nov.

Taxon classificationAnimaliaColeopteraCarabidae

8FD37B09-F284-52A9-B845-7C6BBA2ADD1B

http://zoobank.org/6FDFFBC6-C84B-4070-AEC5-D4D39F730356

Figs 20 , 25 , 35 , 53

###### Material examined.

***Holotype***: New Caledonia • ♂; “21°33'36"S/165°45'20"E NEW CALEDONIA: Prov Sud. Col d’Amieu, 440m el, 4–16.iii.2007 headlamp search”// “EMEC1139863” Holotype pinned, with genitalia in a separate microvial. Specimen relatively well-preserved, but with missing palpomeres, left antennomeres 2–11, right tarsomeres 1–5, left and right mesotibiae and mesotarsomeres 1–5, and left and right metatarsomeres 1–5. Source collection EMEC, deposited MNHN. Type locality as given on label.

###### Diagnosis.

Most similar to *C. magnus*. However, the latter species has striae 2–5 shallowly impressed from near the elytra base to the apex (vs. 2–4 impressed only in the apical 1/3, and 5 in apical 1/2) and apical lamella of the median lobe more widely rounded at tip (Figs 25, 26). Also resembles *C. cyllodinus* but in *C. amieuensis* the eyes are conical, head lacking a notch deep enough to receive the antenna ventral to the eye and the male metafemur does not have a prominent, ventral tooth. The male aedeagus is distinctly different (Fig. 25).

###### Description.

***Habitus*.** Small sized for oodines BL: 7.20 mm, BW: 3.50 mm, with ovate, moderately convex body (Fig. 20). ***Color and luster*.** Dorsal and ventral surface primarily black, with pronotum sides brownish black, fore part of head and femora dark reddish brown, antennomeres 1–3 and tibiae reddish brown, palpi, antennomeres 4–12 and tarsomeres yellowish brown. Integument moderately glossy, without spectral iridescence. ***Microsculpture and punctation*.** Head, pronotum and elytra with isodiametric meshes well impressed, prosternum in front and abdominal ventrites in middle with slightly transverse meshes, prosternal process with isodiametric meshes less impressed than on dorsal surface, rest of ventral surface with scarcely-visible sculpticells or sculpticells not apparent. Micropunctation evident on head, pronotum, prosternum medially (including prosternal process) and medial parts of abdominal ventrites, more or less uniform, lacking on remaining part of ventral surface; abdominal ventrite 1 with some large punctures at sides, ventrites 2–5 at sides moderately rugose. ***Chaetotaxy*.** Labrum with six setae distributed more or less evenly across width each in its own socket. Clypeal setae present. Supraorbital setae absent. Elytron without discal setiferous punctures. Abdominal ventrites 1–5 without ambulatory setae. **Head.** Less than 2 1/2 × as wide as pronotum (Suppl. material 2: Table S1). Eye relatively large, moderately protruded, EyW/HW: 1.47. Labrum anterior margin straight. Frontoclypeal suture present medially, almost obliterated at sides. Antenna moderately long, with last segment not exceeding pronotal base and pubescence starting from segment 4. Mentum tooth with paramedial border distinct. ***Thorax*.** Pronotum ~ 1 2/3 wider than long (PW/PL: 1.70); width at apex less than 2 × at widest point (PW/PA: 2.16). Disc with middle line very fine, without apical transverse impression; anterior angles slightly, widely rounded at tips, anterior margin concave, anterior submarginal sulcus present, interrupted in middle. Prosternal process rhomboidal, finely bordered at apex, not bordered between coxae. Mesosternum anteriorly with two prominent, symmetrical tubercles separated by a space smaller or equal to diameter of one of them. Metepisternum wider than long, with lateral margin barely convex, coadunation with epipleuron long, extended along most of length. **Elytra.** Slightly longer than wide (EL/EW: 1.11). Basal margin forming distinct denticle at shoulder, ended medially just before level of parascutellar puncture. Humeral submarginal carina present. Apical sinuation shallow. Parascutellar striole absent, striae 1–5 present at apex, ended well before basal margin; striae 6 and 7 well impressed throughout, 7 reaching basal margin, 6 ended near but not reaching margin. Elytral intervals 1–6 where evident at apex, convex, interval 7 slightly convex in anterior 1/2, clearly convex in posterior 1/2, interval 8 convex throughout, from 2–3 × narrower than interval 7. Granulation in marginal furrow evident in first 1/5 and last 3/5, lacking in second 1/5. ***Legs*.** Male protarsomeres 1–3 asymmetrically expanded, beneath with adhesive setae (tarsomeres 2 and 3 fully covered, tarsomere 1 covered only on apical 1/4), each of them transverse, wider than long. ***Female genitalia*.** Female specimens unknown. ***Male genitalia*.** Median lobe of aedeagus in lateral view long, curved ventrally, with rather thin apex (Fig. 35); lobe in dorsal view, with apical lamella well bent to left, with left side clearly concave and right side slightly sinuate (Fig. 25); basal bulb rounded dorsally; sclerotized portion of the endophallus with three sclerites, most clearly visible from ventral side.

###### Etymology.

The specific epithet *amieuensis* is based on the type locality Col d’Amieu and is treated as an adjective.

##### Coptocarpus magnus sp. nov.

Taxon classificationAnimaliaColeopteraCarabidae

9AA577AC-3EC0-5766-AC7E-8028158783EA

http://zoobank.org/829A1055-4B5E-4127-BD97-4C4A67C6BF9D

Figs 13 , 26 , 36 , 40 , 51 , 52

###### Material examined.

***Holotype***: New Caledonia • ♂; “NEW CALEDONIA Aoupinié, 20 km NE Poya, 650m 18-19 May1984 G. Monteith & D. Cook”// “Hoplolenus cyllodinus Fvl det. B.P. Moore ‘84” Holotype pinned, with genitalia in a separate microvial. Source collection QM, deposited MNHN. Type locality as given on label.

***Paratype***: New Caledonia • ♀; “NEW CALEDONIA 11159 21°22'Sx165°20'E, Me Maoya Camp, 1150 m. Burwell, Monteith & Wright” [QM].

###### Diagnosis.

Only *C. magnus*, *C. lescheni*, and *C. erwini* have completely impressed elytral stria. Of these three species only *C. magnus* has the apicolateral setae of the clypeus.

###### Description.

***Habitus*.** Medium sized, BL: 9.60 mm and BW: 4.25 mm (paratype, BL: 8.80 mm and BW: 4.20 mm), ovate, slightly convex body. ***Color and luster*.** Head; pronotum, elytra and ventral surface of body dark reddish black; legs deep reddish brown or black, tarsomeres and femur dorsally paler brown or black. Antennae and palpi brown. Integument moderately dull, elytra slightly glossier than head and pronotum, without spectral iridescence except for irregularly on abdominal ventrites. ***Microsculpture and punctation*.** Dorsal surface of head, pronotum, and elytra with prominent isodiametric meshes; ventral surface with evident sculpticells evident except on proepisterna and laterally on abdominal ventrites, on prosternum, medially on all ventrites and throughout ventrite 6 sculpticells isodiametric or somewhat transversely stretched. Head lacking macropunctation or wrinkles on vertex; head, pronotum and elytra with scattered micropunctation, only on elytra do micropunctures and sculpticells form some irregular rosettes (sensu Spence 1983); abdominal ventrites 1 and 2 coarsely punctate, all ventrites very shallowly wrinkled laterally and 3–6 medially smooth. ***Chaetotaxy*.** Labrum with six setae of nearly equal length each in its own socket, lateral setae slightly larger than medial four. Clypeal setae present near apicolateral corners. Single supraorbital seta present over each eye. Elytron without discal setiferous punctures. Abdominal ventrites 1–5 without ambulatory setae. ***Head*.** Approximately 1/3 as wide as pronotum (Suppl. material 2: Table S1). Eye size moderate, eye somewhat protruded, EyW/HW: 1.32 (paratype, 1.33). Labrum anterior margin straight. Frontoclypeal sutures not evident. Antenna short, with last segment not reaching pronotal base and pubescence starting from antennomere 4. Last labial palpomere swollen, blunt at apex, longer than penultimate one. Mentum tooth without paramedial border. ***Thorax*.** Pronotum ~ 1 2/3 wider than long (PW/PL: 1.60 (paratype, 1.71)); width at apex slightly more than twice width at widest point (PW/PA: 2.13 (paratype, 2.22)). Disc with middle line fine, well impressed, and ended well before apical and basal margins, without apical transverse impression; anterior angles produced, rounded triangular, anterior margin shallowly concave, submarginal sulcus present near angles, lacking in middle 1/3. Prosternal process narrowly rounded, throughout bordered. Mesosternum anteriorly with two low, slightly transverse tubercles separated by a space equal to diameter of one of them. Metepisternum width twice its length, with lateral margin slightly sinuate, coadunation with epipleuron along entire length. ***Elytra*.** Slightly longer than wide (EL/EW: 1.20 (paratype, 1.14)). Basal margin forming small, sharp tooth at shoulder, ended medially at level of stria 3, joined stria 3 and parascutellar puncture fovea with a short, shallow striole or this striole lacking. Humeral submarginal carina present. Apical sinuation clearly evident. All elytral striae impressed, clearly impressed in apical 1/2 and 4–7 clearly impressed throughout; striae 1–3 very shallow in basal 1/2, there marked by minute punctures and impressions; parascutellar striole absent. Elytral intervals flat; granulation in marginal furrow discontinuous, interrupted very broadly at midlength. ***Legs*.** Mesotibia slightly, gradually dilated apically. Basomesotarsus and basometatarsus glabrous dorsally; basomesotarsus cylindrical, and with scattered setae, especially laterally. ***Female genitalia*.** Gonocoxite 2 elongate. Spermatheca long, relatively narrow, nearly straight in basal 1/3, coiled in distal 2/3, differentiated to seminal canal and receptaculum; spermathecal gland connected near basal 1/6 of seminal canal. ***Male genitalia*.** Median lobe of aedeagus in lateral view long, notably bent ventrally, with thin, ventrally curved apex (Fig. 36), edge of ostium with produced tooth (Fig. 40); lobe in dorsal view, with apical lamella bent to right, with right side concave and left side straight (Fig. 26); basal bulb forming edge dorsally with evident crest; sclerotized portion of endophallus visible in repose with three sclerites.

###### Etymology.

The specific epithet *magnus* is Latin for large and this refers to the large size of these beetles, larger than all other New Caledonian species in the group. It is treated as an adjective in the nominative singular.

**Figures 49–51.**
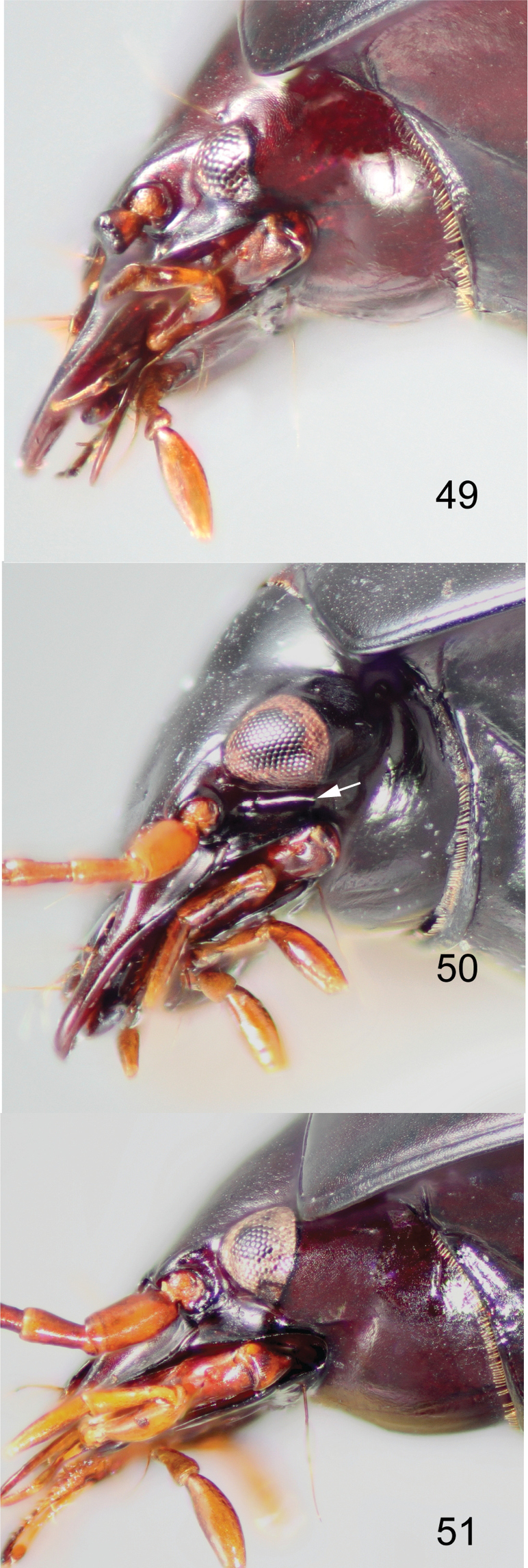
Head in left lateral view of **49***Coptocarpus microps* sp. nov. **50***C. cyllodinus* (Fauvel) **51***C. magnus* sp. nov. Arrow indicates location of notch beneath the eye.

##### *lescheni* group

**Notes.** The only species included in this group possesses three clear autapomorphies: the antennae pubescent from the second segment; the labrum with four setae on anterior margin, with the middle two situated in a shared, small depression; and both the basomesotarsus and the basometatarsus pubescent dorsally. The first and third characters are unique for Oodini.

##### Coptocarpus lescheni sp. nov.

Taxon classificationAnimaliaColeopteraCarabidae

5653B398-D5F0-520E-B5BC-9E5E4332990D

http://zoobank.org/CBB50278-6153-41F3-9241-514FD883AA59

Figs 21 , 52

###### Material examined.

***Holotype***: New Caledonia • ♀; “21°35'11"S/165°46'30"E NEW CALEDONIA: Prov Sud Col D’Amieu, 485 m el, 4–26.iii.2007 Coll. R. Leschen, FITrap NCO78”// “EMEC1139862” Holotype pinned, with reproductive tract in a separate microvial. Specimen not in very good condition, with head separated from the rest of body (but still linked to it by a thin ligament), missing right antennomeres 4–11 and right mesotarsomere 5. Source collection EMEC, deposited MNHN. Type locality as given on label.

###### Diagnosis.

The only species of New Caledonian *Coptocarpus* with dense antennal pubescence starting at antennomere 2, the labrum with four setae, the middle pair very closely set in a small, common depression, and the basomeso- and basometatarsus pubescent dorsally. In all other species the antennae are pubescent from antennomere 4, the labrum has six setae each in its own depression, and the first tarsomeres of the middle and hind tarsi are glabrous dorsally. Note that in the type adjacent to the central pair of setae on the labrum are scarcely apparent structures, which possibly represent additional, very small setae or the vestiges of setae.

###### Description.

***Habitus*.** Small sized, BL: 6.55 mm and BW: 3.10 mm, ovate, moderately convex body (Fig. 21). ***Color and luster*.** Dorsal and ventral surface primarily dark brown, sides of pronotum and abdominal ventrites dark reddish brown; antennae, palpi and tarsi yellowish brown; legs (excluding tarsomeres) dark reddish brown to deep brown. Integument moderately glossy, without spectral iridescence. ***Microsculpture and punctation*.** Dorsal surface of head and pronotum with isodiametric meshes, on head mesh not clearly visible due to strong punctation and wrinkling; elytra with isodiametric meshes on intervals 1–5, with slightly transverse meshes on intervals 6 and 7, and shallow, scarcely visible microsculpture on interval 8; ventral surface mostly with scarcely-visible sculpticells or sculpticells not apparent, only prosternal process with impressed isodiametric meshes evident, and abdominal ventrites with slightly transverse meshes. Head with uniform macropunctation on anterior and middle part, with macropunctation and wrinkles on vertex; pronotum and elytra with punctation smaller and less dense than on head; abdominal ventrites 1 and 2 at sides with several large punctures, ventrites 3–6 at sides with few coarse wrinkles. ***Chaetotaxy*.** Labrum with four setae of equal length each in its own socket, medial two very closely situated, positioned in a small, joint depression. Clypeal setae absent. Supraorbital setae absent. Elytron with one discal setiferous puncture in middle of interval 3 in posterior 1/5 of elytral length. Abdominal ventrites 1–5 without ambulatory setae. ***Head*.** Approximately 2 1/2 × as wide as pronotum (Suppl. material 2: Table S1). Eye small, moderately protruded, EyW/HW: 1.44. Labrum anterior margin straight. Frontoclypeal suture shallow but evident. Antenna moderately long, with last segment not exceeding pronotal base and pubescence starting from segment 2. Last labial palpomere rather swollen, blunt at apex, longer than penultimate one. Mentum tooth with paramedial border. ***Thorax*.** Pronotum 1 2/3 wider than long (PW/PL: 1.65); width at apex more than 2 × less than at widest point (PW/PA: 2.14). Disc with middle line fine and apical transverse impression distinct; anterior angles prominent, rounded at tips, anterior margin concave, anterior submarginal sulcus present near angles, lacking in middle. Prosternal process widely rounded, bordered throughout. Mesosternum anteriorly with two low, symmetrical tubercles separated by a space 4 × longer than diameter of one of them. Metepisternum 1 1/2 × wider than long, with lateral margin convex, coadunation with epipleuron long, located anteriorly and medially. ***Elytra*.** Slightly longer than wide (EL/EW: 1.16). Basal margin forming small denticle at shoulder, ended medially at level between striae 2 and 3. Humeral submarginal carina present. Apical sinuation slight to indistinct. Elytral striae all impressed; parascutellar striole anastomosis with stria 1 ended before basal margin; stria 2 joined to foveate parascutellar puncture; striae 2–4 not reaching basal margin; striae 5 and 6 reaching basal margin; stria 7 distinct medially, nearly reaching basal border. Elytral intervals 1–6 nearly flat, 7 and 8 subconvex to convex; granulation in marginal furrow continuous. ***Legs*.** Mesotibia slightly dilated apically. Basomesostarsus and basometatarsus pubescent dorsally. ***Female genitalia*.** Gonocoxite 2 subtriangular.

###### Etymology.

The specific epithet *lescheni* is treated as a noun in the genitive case and is in honor of Richard Leschen, collector of the type specimen and highly-regarded coleopterist.

###### Notes.

We were not able to recover and examine the internal structures of the female reproductive tract due to the poor condition of these features in the specimen. Apparently, this was due to the fact that most of the tract was everted after death in the aqueous FIT pan solution. Deterioration of the structures made the proper study impossible.

##### Adelopomorpha

Taxon classificationAnimaliaColeopteraCarabidae

Heller, 1916

785255D1-C155-566C-BD99-B3A1C137AFAB

###### Type species.

*Adelopomorpha glabra* Heller, 1916: 270, by original designation.

###### Diagnosis.

Includes species without impressed elytral striae, without submarginal humeral carina, males with protarsomeres 1–3 not expanded and without squamose setae underneath, basomesotarsus ventrally unmodified, and apical lamella of median lobe short, with sides straight or convex on both sides. The narrow male protarsomeres 1–3 is a clear synapomorphy for included species.

###### Redescription.

***Habitus*.** Small sized beetles, BL: 4.45–6.75 mm and BW: 2.30–3.25 mm, rather ovate (Figs 41, 42), very convex body (lateral aspect). ***Chaetotaxy*.** Labrum with six setae of nearly equal length, each in its own socket, lateral setae slightly larger than medial four. Clypeal setae present near apicolateral corners. Single supraorbital seta present over each eye. Anterior seta of stipes present, rather long. Ventral seta of antennomere 2 present, rather long, located in apical 1/2. Pair of long setae on apical margin of ligula. Penultimate labial palpomere glabrous. Mentum with two long, paramedial setae. Submentum with two long, posterolateral setae. Pronotum without setiferous punctures. Elytron without discal setiferous punctures; parascutellar seta present, puncture very large, foveate. Mesocoxa with posteromedial and lateral seta; mesotrochanter with seta. Hind femur without posterior setae. Abdominal ventrites 1–5 without ambulatory setae; last ventrite without or with two setae in male, with four setae in female (female of *A. tethys* and *A. tuberculata* unknown). Gonocoxite 2 with a long dorsomedial seta, and a lateromedial ensiform seta, shorter than the dorsomedial one. ***Head*.** Labrum rectangular, flat, shorter than clypeus, with anterior margin straight or very shallowly emarginate, slightly concave. Antenna short, with last segment not reaching pronotal base and pubescence starting from antennomere 4. Mandible large, long, sharply curved at tip, with sides convex or nearly convex. Apical maxillary palpomere slightly pointed at apex, as long as or slightly longer than penultimate one; last labial palpomere swollen, blunt at apex, longer than penultimate one. Mentum tooth short, triangular, with apex pointed, without or with paramedial border. Gula smooth, somewhat convex. ***Thorax*.** Disc moderately convex, with middle line fine, shallowly or well impressed, and ended well before apical and basal margins, without apical transverse impression, without laterobasal impressions. Anterior angles nearly flat, scarcely convex, anterior margin very shallowly concave, submarginal sulcus impressed along anterior margin near angles, lacking from ~ 1/2–2/3 of middle; basal margin, shallowly sinuate, without bead; lateral bead evident, complete, ended at posterior angles. Prosternum with median longitudinal sulcus distinct, with prosternal process elongate, broadly rounded at apex, bordered throughout. Mesosternum deeply concave, with or without medial tubercle (situated just posterior of mesosternal collar). ***Elytra*.** Humeral submarginal carina absent. Disc convex; no elytral striae impressed, those marked by minute, hardly visible punctures, punctures slightly larger laterally; parascutellar striole absent. Elytral intervals flat; interval 9 transformed in marginal furrow; granulation in marginal furrow discontinuous, interrupted broadly at midlength or in second quarter. ***Legs*.** Metacoxal basal sulcus short, ended at medial 1/3. Mesotibia dilated apically, male mesotibia not more dilated than female; submedial assemblage of mesotibial setae mostly of 2 or 3, rarely 4 setae. Male protarsomeres 1–3 not expanded, elongate, longer than wide, without adhesive setae beneath, each of protarsomeres 2–4 centrically attached to preceding protarsomere. Male basomesotarsus not modified, i.e., with setae small, thin, as in female. Mesotarsomeres 2–4 in both sexes with long and dense setae underneath. ***Female genitalia*.** Gonocoxite 2 subtriangular. Spermatheca elongate; spermathecal gland with long atrium, connected near basal 1/3; common oviduct large, connected to bursa. ***Male genitalia*.** Median lobe of aedeagus in lateral view more or less curved ventrally, tapering to apex; apical lamella (dorsal view) short, with both sides straight or convex, slightly oriented to right to nearly straight; ostium long, reaching basal bulb; basal bulb short, rounded dorsally.

##### *tethys* group

**Notes.** Comprises a single species with isodiametric microsculpture on the elytra and males with two setiferous punctures on ventrite 6.

##### Adelopomorpha tethys sp. nov.

Taxon classificationAnimaliaColeopteraCarabidae

F0F20848-6E53-5F98-AA2C-CD26AAD48868

http://zoobank.org/C4A275B5-DC5E-468D-84A4-23115734DF28

Figs 27 , 37 , 52

###### Material examined.

***Holotype***: New Caledonia • ♂; “NEW CALEDONIA Mandjélia, above Pouébo 11-13 May 1984, 6-750 m G. Monteith & D. Cook” Holotype pinned, with genitalia in a separate microvial. Source collection QM, deposited MNHN. Type locality as given on label.

###### Other material.

New Caledonia • ♂; “21°11'Sx166°01'E: Mt. Koghis, 500 m, 22 Nov 2000 G.B. Monteith. Pyrethrum trunks & logs. 9931” [ZSM].

###### Diagnosis.

Similar to *C. microps* in size and the lack of impressed striae 1–5, but with much larger, prominent eyes and male having protarsomeres 1–3 not expanded. Both *Adelopomorpha glabra* and *A. tuberculata* also lack impressed striae, but are smaller, and in *A. tethys* the male has a pair of setae on ventrite 6 that is lacking in *A. glabra* and *A. tuberculata*. The aedeagus is also decisively different (Fig. 27), with a relatively short and slightly acute tip.

###### Description

**(based on holotype)**. ***Habitus*.** Small sized, BL: 6.75 mm and BW: 3.25 mm. ***Color and luster*.** Head dark reddish black; pronotum, elytra and ventral surface of body black, pronotum marginally diaphanous reddish black, extreme apical edge of ventrite 6 deep reddish black medially; legs deep reddish brown; antennae and palpi brown. Integument moderately dull, without spectral iridescence except ventrally. ***Microsculpture and punctation*.** Dorsal surface of head, pronotum, and elytra with prominent isodiametric meshes; ventral surface with scarcely-visible sculpticells or sculpticells mostly not apparent. Head lacking macropunctation or wrinkles on vertex; head, pronotum and elytra with scattered micropunctation, only on elytra do micropunctures and sculpticells form some irregular rosettes (sensu Spence 1983); all abdominal ventrites very shallowly wrinkled to nearly smooth. ***Head*.** Approximately 1/3 as wide as pronotum (Suppl. material 2: Table S1). Eye size moderate, eye somewhat protruded, EyW/HW: 1.35. Frontoclypeal sutures not evident. Mentum without paramedial border. ***Thorax*.** Pronotum 1 2/3 wider than long (PW/PL: 1.67), with sides evenly, very shallowly rounded from anterior angles to posterior ones; maximal width in posterior 1/5; width at apex 2 2/3 × less than at widest point (PW/PA: 2.32). Posterior angles obtuse, broadly rounded. Mesosternum without medial tubercle. Metepisternum 1 1/3 × wider than long, with lateral margin slightly convex, coadunation with epipleuron along entire length. ***Elytra*.** Broad, nearly as wide as long (EL/EW: 1.06). Basal margin distinct, forming small, rounded callosity at shoulder, ended medially at level of parascutellar punctures, joined to parascutellar puncture fovea by a short, shallow striole. Apical sinuation scarcely evident, very shallow. ***Female genitalia*.** Female specimens unknown. ***Male genitalia*.** Median lobe of aedeagus in lateral view long, almost straight (Fig. 37); lobe in dorsal view with apical lamella moderately long, rounded, triangular and slightly oriented to right (Fig. 27); sclerotized portion of endophallus visible in repose, with two sclerites.

###### Etymology.

The specific epithet *tethys* is treated as a noun in the genitive case and is from the mythological Greek Titan goddess Tethys, said to be the primal source of water to nourish Earth.

###### Notes.

The male specimen from Mt. Koghis (“Additional specimen examined” above) differs from the holotype in somewhat larger eyes (EyW/HW > 1.40), mentum with distinct paramedial border, pronotum with anterior submarginal sulcus present laterally each side and absent in medial 1/4, mesosternum with transversely elongate medial tubercle, and somewhat different structure of the median lobe of aedeagus in lateral view. Given the significant distance (~ 280 km) between the collecting localities and the several morphological differences between them, it is possible that the population from Mt. Koghis represents a separate species, and we exclude this specimen from being a paratype. However, given that these are slight and subtle differences in otherwise very similar looking individuals, it is also possible that this specimen is a conspecific variant. Additional specimens and further study are needed to settle this question.

**Figures 52, 53.**
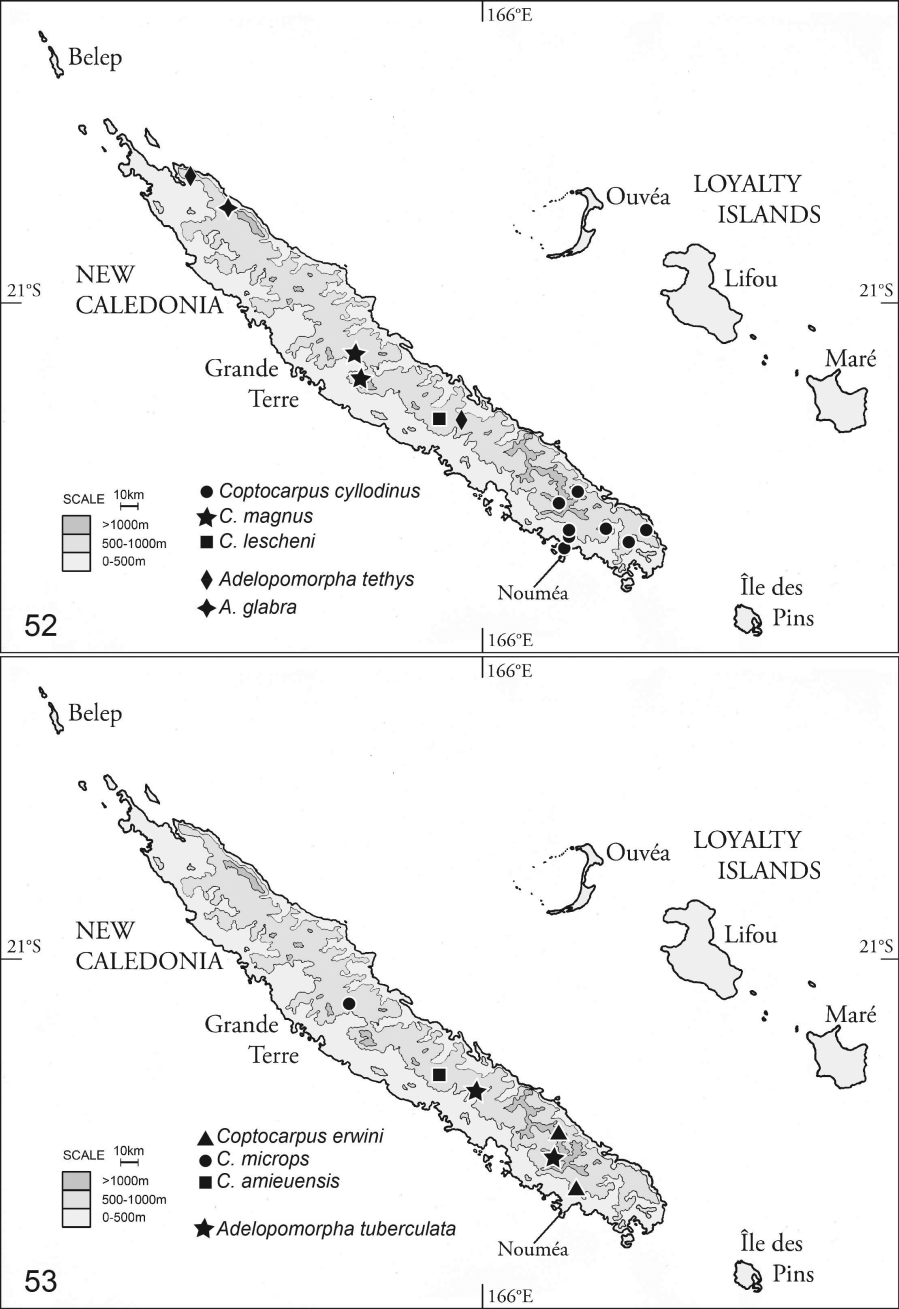
Maps of localities of New Caledonian Oodini.

##### *glabra* group

**Notes.** Comprises species with slightly transverse microsculpture on elytra and males without setigerous punctures on ventrite 6.

##### Adelopomorpha tuberculata sp. nov.

Taxon classificationAnimaliaColeopteraCarabidae

4EFF850B-0A23-5B6E-A046-ADEC98DACFA3

http://zoobank.org/9E94D72E-997A-4550-B6E7-0FED21757F42

Figs 28 , 38 , 41 , 53

###### Material examined.

***Holotype***: New Caledonia • ♂; “NEW CALEDONIA Mt. Do 0.5km below summit, 28Jan2004G.B.Monteith”// “QM berleseate 1111 21°45'Sx166°00'E, rainforest, 950 m, sieved litter” Holotype pinned, with genitalia in a separate microvial. Source collection QM, deposited MNHN. Type locality as given on label.

***Paratypes***: New Caledonia • ♂; “NEW CALEDONIA 11420 21°45'Sx166°00'E, Mt. Do summit, 1000m, 22 Nov 2003, G. Monteith, Pyrethrum, trees & logs.” [QM] • ♂; “NEW CALEDONIA Mt. Dzumac, 800-1000m, 23 May 1984 G. Monteith & D. Cook” [QM].

###### Diagnosis.

This species is nearly indistinguishable externally from *A. glabra* except for the lack of the depressed lateral 1/3 of the pronotum present in *A. glabra* (Fig. 43), and its smaller size. Also, it is smaller than *A. tethys*, whose male has setae on ventrite 6. See diagnosis above. The aedeagus is decisively different (Fig. 28), with a relatively long and moderately, narrowly rounded tip.

###### Description.

***Habitus*.** Very small sized, BL: 4.90 mm and BW: 2.45 mm (range, BL: 4.75–5.00 mm, BW: 2.45–2.60 mm), with rather oval and convex body (Fig. 41). ***Color and luster*.** Head dark reddish brown; pronotum, elytra and ventral surface of body black, pronotum marginally diaphanous reddish black; legs deep reddish brown. Deep reddish black medially at the very apical edge of ventrite 6; antennae and palpi brown. Integument moderately dull, with slight, diffused spectral iridescence dorsally and ventrally. ***Microsculpture and punctation*.** Dorsal surface of head and pronotum with prominent isodiametric meshes; elytra with irregular mesh of more or less transversely stretched sculpticells; ventral surface with scarcely-visible sculpticells on ventrite 6 and prosternum, otherwise sculpticells mostly not apparent. Head lacking macropunctation or wrinkles on vertex; head, pronotum and elytra without micropunctation. All abdominal ventrites very shallowly wrinkled to nearly smooth. ***Head*.** Slightly more than 1/4 as wide as pronotum (Suppl. material 2: Table S1). Eye moderate sized, somewhat protruded, EyW/HW: 1.31. Frontoclypeal sutures not evident. Mentum tooth without paramedial border. ***Thorax*.** Pronotum 1 2/3 wider than long (PW/PL: 1.67 (range, 1.71–1.76)), with sides evenly, very shallowly rounded from anterior angles to posterior ones; maximal width in posterior 1/5; width at apex ~ 1/3 that of widest point (PW/PA: 2.88 (range 2.82–3.00)). Posterior angles ~ right angled, broadly rounded. Mesosternum with single, very prominent, medial tubercle. Metepisternum 1 1/2 × wider than long, with lateral margin straight, coadunation with epipleuron along entire length. ***Elytra*.** Broad, as wide as long (EL/EW: 1.00 (range, 1.00–1.02)). Basal margin distinct, forming small, sharp tooth at shoulder, ended medially at level of parascutellar punctures, joined to parascutellar puncture fovea by a short, shallow striole. Apical sinuation not evident. ***Female genitalia*.** Female specimens unknown. ***Male genitalia*.** Median lobe of aedeagus in lateral view long, almost straight (Fig. 38); lobe in dorsal view with apical lamella moderately long, rounded, triangular and oriented to right (Fig. 28); sclerotized portion of endophallus visible in repose, with two sclerites.

###### Etymology.

The specific epithet *tuberculata* draws attention to the relatively large, median mesosternal tubercle in these beetles. It is treated as an adjective in the nominative singular.

##### Adelopomorpha glabra

Taxon classificationAnimaliaColeopteraCarabidae

Heller, 1916

BDE9B2F4-50A6-5589-9327-0F1F6C61D1D9

Figs 14 , 29 , 39 , 42 , 43 , 52

Adelopomorpha glabra Heller, 1916: 270.

###### Material examined.

***Holotype***: New Caledonia • ♀; card-mounted teneral specimen, with original labels, “Drs. F. Sarasin & J. Roux Neukaledonien Panie Wald” // “Adelop. glabra Typus” // “1914” // “Staatl. Mus. für Tierkunde Dresden” // “Senkenberg Naturhist. Sammlungen Dresden, Museum für Tierkunde (MTD) Museum für Tierkunde Dresden (MTD) MTD-COL-Car-Type-00921” [MTKD]. Type locality. Mt. Panié.

###### Other material.

New Caledonia • ♂; Mt. Panié; 950–1300 m; 14–16.v.1984; G. Monteith & D. Cook; [QM] • ♀; same data as for preceding; [QM] • ♂; 20°34'S, 164°46'E; Mt. Panié track; 1500 m; 9.xi.2001; Pyrethrum, trees & logs; C. Burwell; [QM].

###### Diagnosis.

A glabra group species with depressed lateral 1/3 of the pronotum present (Fig. 43). See diagnosis of *A. tuberculata* and *A. tethys* for characteristics to distinguish those very similar species. The aedeagus is decisively different (Fig. 29), with a relatively long and broadly rounded tip, and with the membranous region of the ostium nearly reaching the apex.

###### Redescription.

***Habitus*.** Small sized, BL: 5.69 mm (range, BL: 5.69–6.00 mm), BW: 2.89 mm (range, 2.83–2.89 mm). ***Color and luster*.** Head and pronotum dark reddish brown to nearly black on pronotal disc; elytra and ventral surface of body black, pronotum marginally diaphanous reddish brown; legs deep reddish brown; Ventrite 6 reddish brown, ventrite 5 black or medially at the very apical edge reddish black; antennae and palpi brown. Integument moderately dull, with diffused spectral iridescence dorsally and ventrally, more prominent on elytra. ***Microsculpture and punctation*.** Dorsal surface of head and pronotum with prominent isodiametric meshes; elytra with somewhat transverse microsculpture; ventral surface with scarcely visible sculpticells or sculpticells mostly not apparent, most apparent on prosternal process. Head lacking macropunctation or wrinkles on vertex; head, pronotum and elytra with extremely fine, scattered micropunctation or lacking such punctures; all abdominal ventrites very shallowly wrinkled laterally and smooth medially. ***Head*.** Approximately 1/3 as wide as pronotum (Suppl. material 2: Table S1). Eye moderate sized, somewhat to moderately protruded, EyW/HW: 1.26 (range, 1.26–1.41; the low value in the type may be due to head position in image). Frontoclypeal sutures not evident. Mentum tooth without paramedial border. ***Thorax*.** Pronotum 1 2/3 wider than long (PW/PL: 1.70 (range, 1.60–1.70)), with sides evenly, very shallowly rounded from anterior angles to posterior ones; maximal width in posterior 1/5; width at apex ~ 1/3 that of widest point (PW/PA: 2.60 (range 2.32–2.60)). Posterior angles ~ right angled, rounded. Mesosternum with single, low medial tubercle. Metepisternum 1 1/3 × wider than long, with lateral margin slightly convex, coadunation with epipleuron along entire length. ***Elytra*.** Broad, as wide as long (EL/EW: 1.06 (range, 1.11–1.15)). Basal margin distinct, forming very sharp tooth at shoulder, ended medially at level of parascutellar punctures, joined to parascutellar puncture fovea by a short, shallow striole. Apical sinuation not evident. ***Female genitalia*.** Bursa copulatrix small in comparison with spermatheca; spermatheca narrow, undifferentiated, coiled at distal 2/3; spermathecal gland connected near basal 1/5–1/4 of spermatheca (Fig. 14). ***Male genitalia*.** Median lobe of aedeagus in lateral view long, shallowly curved (Fig. 39); lobe in dorsal view, with apical lamella, short, rounded, and very slightly oriented to right (Fig. 29); sclerotized portion of endophallus visible in repose, with two sclerites.

##### Unnamed *glabra* group specimens

Figs 30, 31, 44, 45

**Remarks.** Among the New Caledonian oodine specimens examined there are 16 individuals of *Adelopomorpha* from localities across Grande Terre that can be readily separated from *A. glabra* and, at least for males based on the aedeagus, distinguished from *A. tethys* and *A. tuberculata*. Within these 16 specimens, all ten males have a similar form of aedeagus, but with some noticeable variation of the shape in almost every individual (Figs 30, 31, 45). Some variation is also evident in the endophallus when viewed in repose, and also the relative development of sclerites and folds of the endophallus. However, some of the endophallus variation is likely due to the state of preservation and the generally poorly sclerotized aedeagus in these beetles. Initially we hypothesized this could represent a single, island-wide polymorphic species or be grouped in as many as three species. Careful inspection of the external morphological features did not yield any consistent characters that correlated with the aedeagal variation. Reproductive tract dissections for the six female specimens present a different pattern. Two very distinctive spermatheca forms are present (Figs 15, 16, 45) and females of both types are sympatric on Mt. Koghis (Fig. 45). Females, like the males, are externally indistinguishable. Given the female spermathecae are so distinct, we are certain that at least two species are present in the material. However, since the *glabra*-like males and females are indistinguishable externally, beyond recognizing males from females, the male and female specimens were not associated directly when collected, and the female forms are not allopatric, we have no basis to associate any particular males and females. In fact, it is possible that the females could be of *A. tethys* and *A. tuberculata*, as no female specimens were collected in association with those species.

Given this situation, we decided that it was premature to name formally any species from this complex of specimens. All such specimens are herein considered as unnamed members of *Adelopomorpha* that await more extensive collecting and subsequent taxonomic work. As an informal working arrangement that allows for including the specimens in the analysis and presenting our findings, we have placed them in the *glabra* group, in the three subgroups below.

Externally, the male and female specimens from the three subgroups differ from typical *A. glabra* in the pronotum being uniformly convex and curved to the lateral margin or with a very slight (just barely visible) flattening in the lateral 1/5 (Fig. 44), and in the smaller size. The *glabra* group males are also very similar to males of *A. tuberculata* and *A. tethys* in the body size and conformation of the lateral surface of the pronotum, but differ in genital features (see below).

##### *glabra* group males

**Material examined.** New Caledonia • ♂; **Me Maoya**; 21°22'S, 165°20'E; 1150 m; 11–13.xi.2002; hand collecting; [QM] • ♂; same data as for preceding; night collecting ;[QM] • ♂; **Mandjélia**; 20°24'S, 164°32'E; summit; 750 m; 6–7.xi.2001; pyrethrum, trees & logs; C.Burwell & G. Monteith [QM] • ♂; same data as for preceding; *Freycinetia* axils; C.Burwell & G. Monteith; [QM] • ♂; **Mt. Koghis**; 22°10'S, 166°31'E; 500 m; berleseate, sieved litter, rainforest; R. Raven; [QM] • ♂; **Mt. Koghis**; 22°10'28"S, 166°30'48"E; 700 m; 12.iii.2007; under rocks/logs; K. Will; EMEC1075692; [EMEC] • ♂; **Aoupinié**; 21°11'S, 165°19'E; 850 m, 20.xi.2000; pyrethrum, trunks & logs; G. Monteith [QM] • ♂; **Gelima**; 21°36'S, 165°58'E; 7 km S Gelima; 730 m; pyrethrum, trees & logs; C. Burwell & G. Monteith [QM] • ♂; **Plateau de Dogny**; 21°37'15"S, 165°52'29"E; trail to Plateau de Dogny; 870 m; 15.iii.2007; sifted leaf litter; K.Will; EMEC1075687; [EMEC] • ♂; same data as for preceding; EMEC1139864; [EMEC].

**Morphological features.** Differs from typical *A. glabra* males in the apical lamella of the median lobe in dorsal view more acuminate distally, with edge of the ostium more distant from the apex. Both have similar (Figs 30, 31), more proximal position of the main sclerite of the endophallus within the lobe compared to the position in *A. tethys*, *A. tuberculata*, and *A. glabra* (Figs 27–29). The unnamed males are also distinct from males of *A. tuberculata* in that the apical lamella of the median lobe in dorsal view is less asymmetrical and nearly straight compared to the right-oriented form in *A. tuberculata*. Additionally, the sculpticells on the elytra and the presence of setiferous punctures on abdominal ventrite 6 in males of *A. tethys* differentiate them from *glabra* group males.

One of the two specimens from Plateau de Dogny [EMEC1075687] is slightly different from all other glabra group males in having a lighter colored dorsum and legs, and elytral striae more clearly punctiform. The specimen also exhibits somewhat different structure of the aedeagus. Its median lobe in dorsal view is appreciably shorter, with the sclerotized portion of the endophallus represented by two well chitinized sclerites, a broad right-side and a slender left-side. The right-side sclerite is also situated more distally within the lobe than those of other *glabra* group males, but similar to *A. glabra* (Fig. 29).

##### *glabra* group females type 1

**Material examined.** New Caledonia • ♀; **Riviére Bleue**; 22°04'S, 166°38'E; Pourina Track; 900 m; 18.xi.2001; berleseate, sieved litter, rainforest; G.B. Monteith [QM] • ♀; **Mt. Koghis**; 22°10'28"S, 166°30'48"E; 700 m, 12.iii.2007; under rocks/logs; K. Will; EMEC1075693; [EMEC].

**Morphological features.** Spermatheca (Fig. 15) very elongate, larger and longer in comparison with bursa copulatrix and common oviduct, coiled at its distal 3/4, differentiated to narrower seminal canal and wider receptaculum; spermathecal gland with relatively short spermathecal canal, connected near basal 1/5 of seminal canal.

##### *glabra* group females type 2

**Material examined.** New Caledonia • ♀; **Mandjélia**; above Pouébo; 11–13 May 1984; 6–750 m; G. Monteith & D. Cook; [QM] • ♀; **Me Maoya**; 21°22'S, 165°20'E; 1150 m; 11–13.xi.2002; pyrethrum, trees & logs; [QM] • ♀; **Mt. Koghis**; 21°11'S, 166°01'E; 500 m; 22.xi.2000; pyrethrum, trees & logs; G. Monteith; [QM] • ♀; **Ningua**; 21°45'S, 166°09'E; Ningua Reserve camp; 1100 m; 12–13.xi.2001; pyrethrum, trees & logs; C. Burwell & G. Monteith; [QM].

**Morphological features.** Spermatheca (Fig. 16) elongate, relatively short in comparison with bursa copulatrix and common oviduct, nearly straight, undifferentiated; spermathecal gland with rather long spermathecal canal, connected near basal 1/3 of seminal canal.

### Key to species of New Caledonian Oodini

**Table:** 

1	Elytra with submarginal carina near humeri (Fig. 47)	**2**
–	Elytra without submarginal carina near humeri (Fig. 46)	**5**
2	Lacking a deep notch below eye (Fig. 51); eye and orbit prominent, rounded and conical; male metafemur without a ventral tooth (male unknown in one species)	**3**
–	Deep notch below eye (Fig. 50); eye and orbit very prominent, almost shelf-like; male metafemur with large, ventral tooth (Fig. 48)	***Coptocarpus cyllodinus* (Fauvel) comb. nov.**
3	Antennae pubescent from antennomere 4; labrum with six setae distributed on anterior margin, each in its own puncture; dorsum of head smooth to very finely punctate posteriorly	**4**
–	Antennae pubescent from antennomere 2; labrum with four setae apparent, the middle pair very closely set in a single depression; dorsum of head coarsely punctate posteriorly	***Coptocarpus lescheni* sp. nov.**
4	Elytral striae 2–4 impressed only in the apical 1/3	***Coptocarpus amieuensis* sp. nov.**
–	Elytral striae 2–4 evidently, though shallowly, impressed from near the elytra base to the apex	***Coptocarpus magnus* sp. nov.**
5	Elytra lacking impressed discal striae, at most striae are slightly indicated by shallow punctures; one supraorbital seta	**6**
–	Elytra with all striae impressed; supraorbital setae lacking	***Coptocarpus erwini* sp. nov.**
6	Development of the eye typical for New Caledonian oodine, eye width clearly more than twice the distance from the anterior edge of the eye to the antennal socket (Figs 50, 51); antennomeres 4–10 length not more than twice the antennomere width; male with protarsomeres 1–3 not expanded	**7**
–	Eye small and rather flat (Fig. 49), eye width ~ equal to or less than twice the distance from the anterior edge of the eye to the antennal socket; antennomeres 4–10 elongate, length ~ 3.5 × the antennomere width; male protarsomeres 1–3 asymmetrically expanded	***Coptocarpus microps* sp. nov.**
7	Elytra slightly glossier than pronotum and with spectral iridescence evident at least in the apical 1/3; males lacking setiferous punctures on ventrite 6	**8**
–	Elytra not glossier than pronotum and without spectral iridescence; males with a pair of setiferous punctures on ventrite 6	***Adelopomorpha tethys* sp. nov.**
8	Female specimens (with two pairs of setiferous punctures on ventrite 6)	**9**
–	Male specimens (without setiferous punctures on ventrite 6)	**10**
9	Pronotum noticeably depressed in the lateral 1/3 of the pronotum (very slightly and best viewed by shifting light over the surface) (Fig. 43). Size (5.7–6.0 mm)	***Adelopomorpha glabra* Heller**
–	Pronotum uniformly convex and curved to the lateral margin, or with a very slight (just barely visible) flattening in the lateral 1/5 (Fig. 44). Size (5.2–5.6 mm, one specimen 5.9 mm)	***Adelopomorpha tuberculata* sp. nov.** or ***glabra* group females types 1 and 2**
10	Pronotum with noticeable (though very slight) flattening or depression of the lateral 1/3 evident (Fig. 43). Size (5.7–6.0 mm). Aedeagus symmetrical in dorsal view with ostium ended very near tip (Fig. 29)	***Adelopomorpha glabra* Heller**
–	Pronotum uniformly convex and curved to the lateral margin, or with a very slight (just barely visible) flattening in the lateral 1/5 (Fig. 44). Size (4.5–5.7 mm). Aedeagus not as above (Figs 28, 30, 31)	**11**
11	Aedeagus distinctly deflected to the right in dorsal view, tip somewhat narrowly rounded and slightly thick in lateral view (Fig. 28)	***Adelopomorpha tuberculata* sp. nov.**
–	Aedeagus symmetrical, or nearly so, in dorsal view, tip somewhat narrowly or moderately broadly rounded and thin in lateral view (Figs 30, 31, 45)	***glabra* group males**

## Discussion

### Implications of this study for the classification of Oodini and Thryptocerina

Erwin (1974: 1) specified that one of the main goals in his work on the genus *Coptocarpus* was “to provide notes on the African-Australian relationships between *Coptocarpus* and the so-called “Thryptocerini” (Jeannel, 1949).” Erwin (ibid.) stated that among the Oodini only *Coptocarpus*, *Thryptocerus*, *Orthocerodus*, *Hoplolenus*, and *Holcocoleus* species have the male anterior tarsi “asymmetrically arranged,” referring to the asymmetrical expansion of the tarsomeres relative to the axis of attachment of the tarsomeres to each other. Among those genera he correctly notes that *Holcocoleus* species have the least asymmetrically developed tarsomeres and are the only taxa that possess the ambulatory setae on the abdominal ventrites. Based on his study, Erwin concluded (in “Phylogenetic and Zoogeographic Notes,” ibid.) that the “striking similarities between the Australasian *Coptocarpus* and the African “Thryptocerini’ are more than convergences.” It is notable that the Indian genus *Holcocoleus* is not included in his list of prospective thryptocerine taxa, but is proposed as a possible “key” to understanding the group, and therefore in need of further study. In this regard, our study picked up where Erwin left off. Our results support a monophyletic Thryptocerina, exclusive of *Holcocoleus* and including the New Caledonian taxa not studied by Erwin. The relationship of Thryptocerina to other Oodini remains an open question not fully addressed by the outgroup taxa included here.

Thryptocerina is supported by two unambiguous synapomorphic state changes, the elongate form of prosternal process (character 14, state 2) and the loss of the ambulatory setae on the abdominal ventrites (character 24, state 0), and one state change that only provides support under an accelerated transformation hypothesis, the presence of the parascutellar setiferous puncture (character 11, state 0). We found no evidence of a close affiliation of *Holcocoleus* to any thryptocerine taxa and it did not turn out that this genus was the “key” to understanding the group contrary to Erwin’s prediction. Further evidence of apomorphic dissimilarity for *Holcocoleus* is apparent in the combination of a straight or nearly straight receptaculum and spermathecal gland attached to apical 1/2 of seminal canal (character 35, state1; character 36, state 0), a unique combination for taxa we examined. On the other hand, *Evolenes* is clearly placed as sister to Thryptocerina by five unambiguous, albeit homoplasious characters (Fig. 17). Given that *Evolenes* is found only in the New World, this opens the possibility of a northern Gondwanan connection (amphi-Atlantic) (Will 2020b) and would be consistent with the genus representing a Gondwanan relict in the present-day Nearctic biota.

Our results also suggest that within Thryptocerina there remain phylogenetic and taxonomic questions, though there are some significant implications to be drawn from the phylogeny. The subtribe splits into two major clades, the *Adelopomorpha* clade and a clade of *Hoplolenus*-like taxa. Most taxa in the latter clade are currently placed in *Coptocarpus*, but the nomenclaturally oldest genus in the clade is *Hoplolenus* and we use that taxon to refer to the clade, but refrain from making sweeping nomenclatural changes until a more inclusive study of Australian species is conducted. Very notable synapomorphies for the taxa of *Hoplolenus* clade are found in the manner by which the male protarsomeres 3 and 4 insert into 2 and 3, and chaetotaxic patterns. The insertion is clearly asymmetrical relative to the expanded tarsomere width (character 18, state 1), and the protarsomere ventral, squamose setae are present on apical 2/3 of ventral surface (character 19, states 2, 3), these states in both characters also being shared with *Evolenes*.

Adelphotaxon to all remaining OTUs in *Hoplolenus* clade is a newly discovered and currently unnamed member of *Coptocarpus* from Mt. Lewis, QLD, Australia. This is tentatively placed as belonging to a new, sixth species group, as it has a different combination of character states, distinct from any of Erwin’s species groups. Given its unique characteristics and phylogenetic position, it is possible that it merits generic status. However, any definitive conclusion depends on a broader treatment of the Australian species.

Erwin (1974) stated that “the members of the *philipi* and *chimbu* groups are most similar to *Orthocerodus* and *Hoplolenus* members, respectively.” Material of the *chimbu* group was not available for study and thus we are unable resolve its taxonomic position in Thryptocerina. The cladistic analysis placed *C. philipi* Erwin, 1974 as a separate lineage within the *Hoplolenus* clade, as the sister to the clade containing the African *Hoplolenus*, Malagasy *Thryptocerus* and *Orthocerodus*, and a clade of the remaining species of *Coptocarpus* and *Lobatodes*.

As presently conceived, paraphyly of *Coptocarpus* is clear. This result is not unexpected because the genus, as was treated by Erwin, included species that share many plesiomorphic states but no apparent synapomorphies to support monophyly exclusive of other thryptocerine genera. Whereas the placement of *Hoplolenus*, *Thryptocerus* and *Orthocerodus* is consistent with previous studies, the inclusion of *Lobatodes* was quite unexpected and strikes us as debatable. *Lobatodes decellei* is placed in an unresolved clade with three species of Australian *Coptocarpus* representing the *grossus*, *chaudoiri*, and *australis* species groups. Two characters support this clade in all trees; the elytral anterior discal punctures are present (character 12 state 0) and the spermatheca is elongate and differentiated into seminal canal and receptaculum (character 33 state 1). Presence of the elytral anterior discal punctures is also found in the outgroup taxa *Simous* and *Holcocoleus*, and is independently gained in this clade and in *C. erwini*. Character 33, the elongate and differentiated spermatheca, is also found in the outgroup taxa *Simous* and *Holcocoleus* and is independently gained in this clade, *glabra* group female type 2, and *C. magnus*.

While *Lobatodes* is placed with this group of Australian *Coptocarpus* in our analysis by these few, homoplasious characters, its distinct autapomorphic character states distinguish it from all other taxa in the polytomy. The setose penultimate labial palpomere and nearly straight shape of spermatheca, with a specific, balloon-like receptaculum (Fig. 2), the form of the median lobe, and protarsomere setation (Characters 6, 19, 26, 29, and 35) make this an unexpected result. In particular, all New Caledonian *Coptocarpus* and the Australian *grossus*, *chaudoiri*, *australis* species groups share both a distinctive form and a distinctive length of the apical lamella of the median lobe (Characters 26 and 29, respectively, Figs 22–26) that are not present in *Lobatodes*. We suspect *Lobatodes* is unlikely to be a close relative of the Australian *Coptocarpus* it is placed with. At the moment, we retain the genus *Lobatodes* and refrain from making any classificatory changes until a fuller analysis is completed. *Lobatodes* notwithstanding, all the newly described New Caledonian *Coptocarpus* and the Australian *grossus*, *chaudoiri*, *australis* species groups form a clade that we recognize as *Coptocarpus*.

*Adelopomorpha* and *C.* sp. group 6 share a plesiomorphic medial lobe of aedeagus that is straight or convex on both sides in dorsal view (character 27, state 0), but *Adelopomorpha* is supported as a clade by several state changes including the absence of impressed elytral striae (character 8, state 2) and the complete lack of modifications of the male protarsomeres (character 16, state 1; character 19, state 3; and character 20, state 1). The synapomorphic support for this genus as sister to the *Hoplolenus* clade allows for definitive recognition of the generic placement of the species of *Adelopomorpha* described herein.

### Evolution of the female reproductive tract

The variation among the studied oodine taxa (Bousquet 1996; Guéorguiev and Liang 2020; present study) demonstrates that evolution of the female reproductive tract system is rather complicated; basically, the position and configuration of the female spermatheca varies widely. The ancestral state of the female spermatheca of thryptocerines is hypothesized to be a long form, apically twisted, rather than coiled, with the spermatheca joining the reproductive tract via a seminal canal placed near the juncture of the common oviduct and the bursa (Figs 10, 12, 14). In this configuration, the spermathecal gland the seminal canal in its basal 1/4 and the bursa has no dorsal lobe. This combination of states is found in *Adelopomorpha* and the Australasian members of the *Hoplolenus* clade, with the notable exception of *Lobatodes*. Further diversification of the female tract appears to have involved evolution into different directions. The female tract configuration in *Adelopomorpha* exhibits two state combinations; first, the ancestral spermathecal configuration is retained but the bursa is decreased appreciably in size (Figs 14, 15); second, the bursa retains its dimensions, but the spermatheca is significantly shortened (Fig. 16). In both variations, the position of the spermathecal gland on the seminal canal remained unchanged. Within *Coptocarpus*, the spermathecal configuration varies more or less markedly. In *C.* sp. group 6, *C. cyllodinus* and *C. magnus* it is very near the plesiomorphic condition with the apical 1/2 of the spermatheca twisted (Fig. 10) or coiled (Figs 12, 13), and the bursa may be with or without a dorsal lobe. *Coptocarpus erwini* has a spermathecal configuration very similar to that of Australian *C.* sp. group 6, but with a gland attachment on the seminal canal in medial 1/3 that represents an apomorphic condition (Fig. 11).

The female reproductive tract in Australian *grossus*, *chaudoiri*, and *australis* species groups appears to be a highly specialized configuration, with two synapomorphies not found in *Lobatodes*, again suggesting that this genus is not closely related. Species of the three Australian groups have an inflated spermatheca, extraordinarily large relative to the bursa copulatrix and common oviduct, with the distal 2/3–3/4 (receptaculum itself) spiraled and progressively enlarged compared with proximal 1/4–1/3 (seminal canal) narrowed (Figs 7–9). An extreme case of this condition exists in *C. grossus*, in which the seminal canal and bursa have the internal walls distinctly chitinized (notable cuticular thickening). Members of these three Australian species groups also share a significantly shortened common oviduct that broadly joins with the spermatheca forming a common connection with the bursa (see Figs 7, 8; common oviduct broken in Fig. 9). The New Caledonian *C. magnus* has a spiral receptaculum similar to the taxa above, but its appearance is different, with the apical 1/3 of the spermatheca increasingly narrower than the medial 1/3 (Fig. 13).

The female tract configuration of the clade including *Thryptocerus*, *Orthocerodus*, and *Hoplolenus* is significantly different from what is found in its sister clade composed of *Coptocarpus* species. Species of *Hoplolenus* have a relatively large bursa copulatrix that is cup-shaped not extended distally (compare Figs 5 and 6), a well-developed common oviduct, and a short, globular (sessile) receptaculum that is attached to the bursa close to the attachment point of the common oviduct. *Thryptocerus agaboides* exhibits a very distinct condition, with the bursa having the distal lobe and a sessile receptaculum attached on the common oviduct (and not on bursa) so that the latter seems to be a growth from or fusion with the former. The sessile receptaculum is likely a synapomorphy for this clade and although females of *Orthocerodus* were not available for study, we predict they will exhibit a similar configuration.

## Supplementary Material

XML Treatment for Thryptocerina

XML Treatment for Coptocarpus

XML Treatment for Coptocarpus microps

XML Treatment for Coptocarpus erwini

XML Treatment for Coptocarpus cyllodinus

XML Treatment for Coptocarpus amieuensis

XML Treatment for Coptocarpus magnus

XML Treatment for Coptocarpus lescheni

XML Treatment for Adelopomorpha

XML Treatment for Adelopomorpha tethys

XML Treatment for Adelopomorpha tuberculata

XML Treatment for Adelopomorpha glabra

## Supplementary materials

Supplementary material 1 Thryptocerine morphology matrix with trees Data type Morphological matrix file and trees Explanation note Complete thryptocerine morphology matrix file in nexus (text) format with trees. Can be opened with text editors but intended for use with Mesquite and similar programs. https://binary.pensoft.net/file/554063This dataset is made available under the Open Database License (http://opendatacommons.org/licenses/odbl/1.0/). The Open Database License (ODbL) is a license agreement intended to allow users to freely share, modify, and use this Dataset while maintaining this same freedom for others, provided that the original source and author(s) are credited.Kipling Will, Borislav Guéorguiev

Supplementary material 2 Table S1. Measurements and ratios Data type Morphology specimen measurements Explanation note Excel file of raw and converted measurements for all specimens. https://binary.pensoft.net/file/554064This dataset is made available under the Open Database License (http://opendatacommons.org/licenses/odbl/1.0/). The Open Database License (ODbL) is a license agreement intended to allow users to freely share, modify, and use this Dataset while maintaining this same freedom for others, provided that the original source and author(s) are credited.Kipling Will, Borislav Guéorguiev

Supplementary material 3 Morphology matrix in Excel format Data type Morphological matrix file Explanation note Verbose complete morphology matrix in an Excel table. https://binary.pensoft.net/file/554065This dataset is made available under the Open Database License (http://opendatacommons.org/licenses/odbl/1.0/). The Open Database License (ODbL) is a license agreement intended to allow users to freely share, modify, and use this Dataset while maintaining this same freedom for others, provided that the original source and author(s) are credited.Kipling Will, Borislav Guéorguiev

Supplementary material 4 TNT IENUM Analysis files Data type TNT Analysis files Explanation note All input and output files for TNT implicit enumeration analysis combined into a single PDF. https://binary.pensoft.net/file/554066This dataset is made available under the Open Database License (http://opendatacommons.org/licenses/odbl/1.0/). The Open Database License (ODbL) is a license agreement intended to allow users to freely share, modify, and use this Dataset while maintaining this same freedom for others, provided that the original source and author(s) are credited.Kipling Will, Borislav Guéorguiev

Supplementary material 5 TNT Heuristic Analysis files Data type TNT Analysis files Explanation note All input and output files for TNT heuristic analysis as a single PDF. https://binary.pensoft.net/file/554067This dataset is made available under the Open Database License (http://opendatacommons.org/licenses/odbl/1.0/). The Open Database License (ODbL) is a license agreement intended to allow users to freely share, modify, and use this Dataset while maintaining this same freedom for others, provided that the original source and author(s) are credited.Kipling Will, Borislav Guéorguiev
