# Supplementary material for: Phylogenetic systematics of the genera of Thryptocerina Jeannel, 1949 and new species from New Caledonia (Coleoptera, Carabidae, Oodini)
Source: Zookeys. 2021 Jun 16;1044:375–425. doi: 10.3897/zookeys.1044.63775 (PMC8222308; doi:10.3897/zookeys.1044.63775)
Supplement: Supplementary material 4 — TNT IENUM Analysis files [file zookeys-1044-375-s004.pdf]

```
xread
36 24
t0    010111000000010000000010001100011000
t1    00011010001111002120000000?01000001
t2    000111[0 1]00010010010001000000000001110
t3    01201100000112000120010100101211121?2
t4    000110000100020001100101001002001111
t5    0121110201011 10???????01?????20121?2
t6    1111110201011200011001?100101???????
t7    010011[0 1]001011200?1200111001?0001????
t8    000111000100020021200011021012111101
t9    000111000100000001200101021112101101
t10   000111000100120001200111021012001101
t11   010111000001120021201011000000010001
t12   000001011101110011200111021110010001
t13   111001[0 1]01001002???????11?????001????
t14   0001010111011120012 00??102101???????
t15   000101001101111200120011102111101 1001
t16   000001000100021001200110021111010000
t17   000111[0 1]201011210012001?102101???????
t18   0001111201011211203110111 10?00000001
t19   00011102010112[0 1]1203110?101000???????
t20   0001111201011211203110?111000???????
t21   0001111201011211203110?111000???????
t22   000111120101121???????11?????0000111
t23   000111120101121???????11?????0001101
;
```

Reading from data.ss
Matrix (36x24, 16 states). Memory required for data: 0.08 Mbytes
Again reading from TNTCommands.txt
Tree file: TNT_Trees.txt
Randomizing insertion sequence in Wagner trees
Space for 10000 trees in memory
0 uninformative characters are inactive
Implicit enumeration, 30 trees found, score 108.
30 trees saved to TNT_Trees.txt

Trees acquired from TNT using Mesquite's Zephyr package.
TNT run on SSH Server
Analysis started Fri Jan 22 12:46:40 PST 2021
User on originating (local) computer: Loxandrus

Taxa: Taxa
Matrix: Character Matrix
Number of taxa analyzed: 24

Remote computer on which analysis was conducted: Carabid at UCB (carabid.espm.berkeley.edu) via SSH
Username on remote computer: kipwill
Directory on remote computer holding working directory: /home/kipwill/
Name of working directory on remote computer : TNT-2021.01.22.1

```
cd \/home\/kipwill\/TNT-2021.01.22.1

nohup  "/home/kipwill/tnt" bground  proc TNTCommands.txt && rm -f \/home\/kipwill\/TNT-2021.01.22.1\/running &
```

t0	Simous annamita
t1	Evolenes exarata
t2	Holcocoleus spp
t3	Hoplolenus spp
t4	Lobatodes decellei
t5	Thryptocerus agaboides
t6	Orthocerodus parallelus
t7	Coptocarpus philipi
t8	Coptocarpus grossus
t9	Coptocarpus cf chaudoiri
t10	Coptocarpus thoracicus
t11	Coptocarpus sp group 6
t12	Coptocarpus cyllodinus
t13	Coptocarpus lescheni
t14	Coptocarpus amieuensis
t15	Coptocarpus magnus
t16	Coptocarpus erwini
t17	Coptocarpus microps
t18	Adelopomorpha glabra
t19	Adelopomorpha tethys
t20	Adelopomorpha tuberculata
t21	Adelopomorpha glabra complex males
t22	Adelopomorpha glabra complex 1 females
t23	Adelopomorpha glabra complex 2 females

```
tread 'tree(s) from TNT, for data in data.ss'
(0 (2 (1 ((11 (7 ((3 (5 6 ))((10 (8 (4 9 )))(17 (16 (14 (15 (12 13 )))))))))(19 ((20 (18 21 ))(22 23 )))))))*
(0 (2 (1 ((11 (7 ((3 (5 6 ))((10 (8 (4 9 )))(17 (16 (14 (15 (12 13 )))))))))(19 ((18 21 )(23 (20 22 ))))))))*
(0 (2 (1 ((11 (7 ((3 (5 6 ))((10 (8 (4 9 )))(17 (16 (14 (15 (12 13 )))))))))(19 ((18 21 )(20 (22 23 ))))))))*
(0 (2 (1 ((11 (7 ((3 (5 6 ))((10 (8 (4 9 )))(17 (16 (14 (15 (12 13 )))))))))(19 (20 (18 (23 (21 22 )))))))))*
(0 (2 (1 ((11 (7 ((3 (5 6 ))((10 (8 (4 9 )))(17 (16 (15 (13 (12 14 )))))))))(19 ((20 (18 21 ))(22 23 )))))))*
(0 (2 (1 ((11 (7 ((3 (5 6 ))((10 (8 (4 9 )))(17 (16 (15 (13 (12 14 )))))))))(19 ((18 21 )(23 (20 22 ))))))))*
(0 (2 (1 ((11 (7 ((3 (5 6 ))((10 (8 (4 9 )))(17 (16 (15 (13 (12 14 )))))))))(19 ((18 21 )(20 (22 23 ))))))))*
(0 (2 (1 ((11 (7 ((3 (5 6 ))((10 (8 (4 9 )))(17 (16 (15 (13 (12 14 )))))))))(19 (20 (18 (23 (21 22 )))))))))*
(0 (2 (1 ((11 (7 ((3 (5 6 ))((8 (10 (4 9 )))(17 (16 (14 (15 (12 13 )))))))))(19 ((20 (18 21 ))(22 23 )))))))*
(0 (2 (1 ((11 (7 ((3 (5 6 ))((8 (10 (4 9 )))(17 (16 (14 (15 (12 13 )))))))))(19 ((18 21 )(23 (20 22 ))))))))*
(0 (2 (1 ((11 (7 ((3 (5 6 ))((8 (10 (4 9 )))(17 (16 (14 (15 (12 13 )))))))))(19 ((18 21 )(20 (22 23 ))))))))*
(0 (2 (1 ((11 (7 ((3 (5 6 ))((8 (10 (4 9 )))(17 (16 (14 (15 (12 13 )))))))))(19 (20 (18 (23 (21 22 )))))))))*
(0 (2 (1 ((11 (7 ((3 (5 6 ))((8 (10 (4 9 )))(17 (16 (15 (13 (12 14 )))))))))(19 ((20 (18 21 ))(22 23 )))))))*
(0 (2 (1 ((11 (7 ((3 (5 6 ))((8 (10 (4 9 )))(17 (16 (15 (13 (12 14 )))))))))(19 ((18 21 )(23 (20 22 ))))))))*
(0 (2 (1 ((11 (7 ((3 (5 6 ))((8 (10 (4 9 )))(17 (16 (15 (13 (12 14 )))))))))(19 ((18 21 )(20 (22 23 ))))))))*
(0 (2 (1 ((11 (7 ((3 (5 6 ))((8 (10 (4 9 )))(17 (16 (15 (13 (12 14 )))))))))(19 (20 (18 (23 (21 22 )))))))))*
(0 (2 (1 ((11 (7 ((3 (5 6 ))((10 (4 (8 9 )))(17 (16 (14 (15 (12 13 )))))))))(19 ((20 (18 21 ))(22 23 )))))))*
(0 (2 (1 ((11 (7 ((3 (5 6 ))((10 (4 (8 9 )))(17 (16 (14 (15 (12 13 )))))))))(19 ((18 21 )(23 (20 22 ))))))))*
(0 (2 (1 ((11 (7 ((3 (5 6 ))((10 (4 (8 9 )))(17 (16 (14 (15 (12 13 )))))))))(19 ((18 21 )(20 (22 23 ))))))))*
(0 (2 (1 ((11 (7 ((3 (5 6 ))((10 (4 (8 9 )))(17 (16 (14 (15 (12 13 )))))))))(19 (20 (18 (23 (21 22 )))))))))*
(0 (2 (1 ((11 (7 ((3 (5 6 ))((10 (4 (8 9 )))(17 (16 (15 (13 (12 14 )))))))))(19 ((20 (18 21 ))(22 23 )))))))*
(0 (2 (1 ((11 (7 ((3 (5 6 ))((10 (4 (8 9 )))(17 (16 (15 (13 (12 14 )))))))))(19 ((18 21 )(23 (20 22 ))))))))*
(0 (2 (1 ((11 (7 ((3 (5 6 ))((10 (4 (8 9 )))(17 (16 (15 (13 (12 14 )))))))))(19 ((18 21 )(20 (22 23 ))))))))*
(0 (2 (1 ((11 (7 ((3 (5 6 ))((10 (4 (8 9 )))(17 (16 (15 (13 (12 14 )))))))))(19 (20 (18 (23 (21 22 )))))))))*
(0 (2 (1 ((11 (7 ((3 (5 6 ))((10 (8 (4 9 )))(17 (16 (14 (15 (12 13 )))))))))(19 (18 (23 (22 (20 21 )))))))))*
(0 (2 (1 ((11 (7 ((3 (5 6 ))((10 (8 (4 9 )))(17 (16 (15 (13 (12 14 )))))))))(19 (18 (23 (22 (20 21 )))))))))*
(0 (2 (1 ((11 (7 ((3 (5 6 ))((8 (10 (4 9 )))(17 (16 (14 (15 (12 13 )))))))))(19 (18 (23 (22 (20 21 )))))))))*
(0 (2 (1 ((11 (7 ((3 (5 6 ))((8 (10 (4 9 )))(17 (16 (15 (13 (12 14 )))))))))(19 (18 (23 (22 (20 21 )))))))))*
(0 (2 (1 ((11 (7 ((3 (5 6 ))((10 (4 (8 9 )))(17 (16 (14 (15 (12 13 )))))))))(19 (18 (23 (22 (20 21 )))))))))*
(0 (2 (1 ((11 (7 ((3 (5 6 ))((10 (4 (8 9 )))(17 (16 (15 (13 (12 14 )))))))))(19 (18 (23 (22 (20 21 )))))))));
proc-;
```

```
   mxram 2000;
   report+0/1/0;
   log log.out;
   p data.ss;
   tsave *TNT_Trees.txt;
   rseed[;
   hold 10000;
   xinact;
IENUM;

   save;
   log/;
   tsave/;
   quit;
```
