# Supplementary material for: Phylogenetic systematics of the genera of Thryptocerina Jeannel, 1949 and new species from New Caledonia (Coleoptera, Carabidae, Oodini)
Source: Zookeys. 2021 Jun 16;1044:375–425. doi: 10.3897/zookeys.1044.63775 (PMC8222308; doi:10.3897/zookeys.1044.63775)
Supplement: Supplementary material 5 — TNT Heuristic Analysis files [file zookeys-1044-375-s005.pdf]

```
Reading from C:\Users\Loxandrus\Desktop\Projects\Oodines\TNT-2021.01.22-Run.3\data.ss
Matrix (36x24, 16 states). Memory required for data:   0.07 Mbytes



-----------------------------------------------------------------

   T. N. T.  Version 1.5 (May 2018) - 32 bits (for Windows)

   Copyright (c) 2001  P.A.Goloboff, J.S.Farris, K.C.Nixon

-----------------------------------------------------------------
Again reading from C:\Users\Loxandrus\Desktop\Projects\Oodines\TNT-2021.01.22-Run.3\TNTCommands.txt
Tree file: TNT_Trees.txt
Randomizing insertion sequence in Wagner trees
Space for 10000 trees in memory
0 uninformative characters are inactive
Repl. Algor.     Tree        Score       Best Score   Time       Rearrangs.
100   FUSE     100           108         108          0:00:00    29,362,130
100   FUSE     100           ------      108          0:00:00    29,362,130
Completed search.
Total rearrangements examined: 29,362,130.
No target score defined.  Best score hit 1 times.
Best score: 108.  24 trees retained.
Start swapping from 24 trees (score 108)...
Repl. Algor.     Tree        Score       Best Score   Time       Rearrangs.
---   TBR      29 of 30     ------      108          0:00:00    152,458
Completed TBR branch-swapping.
Total rearrangements examined: 152,458.
Best score (TBR): 108.  30 trees found.
30 trees saved to TNT_Trees.txt
```

```
tread 'tree(s) from TNT, for data in C:\Users\Loxandrus\Desktop\Projects\Oodines\TNT-2021.01.22-Run.3\data.ss'
(0 (2 (1 ((11 (7 ((3 (5 6 ))((10 (8 (4 9 )))(17 (16 ((12 13 )(14 15 ))))))))(19 (18 (21 (20 (22 23 )))))))))*
(0 (2 (1 ((11 (7 ((3 (5 6 ))((10 (4 (8 9 )))(17 (16 (14 (15 (12 13 )))))))))(19 (21 (18 (20 (22 23 )))))))))*
(0 (2 (1 ((11 (7 ((3 (5 6 ))((10 (8 (4 9 )))(17 (16 (15 (14 (12 13 )))))))))(19 (20 (21 (18 (22 23 )))))))))*
(0 (2 (1 ((11 (7 ((3 (5 6 ))((8 (10 (4 9 )))(17 (16 (15 (13 (12 14 )))))))))(19 (18 (23 (21 (20 22 )))))))))*
(0 (2 (1 ((11 (7 ((3 (5 6 ))((8 (10 (4 9 )))(17 (16 ((12 13 )(14 15 ))))))))(19 (20 (18 (21 (22 23 )))))))))*
(0 (2 (1 ((11 (7 ((3 (5 6 ))((8 (10 (4 9 )))(17 (16 (14 (15 (12 13 )))))))))(19 ((18 21 )(22 (20 23 ))))))))*
(0 (2 (1 ((11 (7 ((3 (5 6 ))((10 (4 (8 9 )))(17 (16 (15 (13 (12 14 )))))))))(19 (21 (18 (22 (20 23 )))))))))*
(0 (2 (1 ((11 (7 ((3 (5 6 ))((8 (10 (4 9 )))(17 (16 (14 (15 (12 13 )))))))))(19 (21 ((18 20 )(22 23 ))))))))*
(0 (2 (1 ((11 (7 ((3 (5 6 ))((10 (8 (4 9 )))(17 (16 (15 (13 (12 14 )))))))))(19 (21 (18 (22 (20 23 )))))))))*
(0 (2 (1 ((11 (7 ((3 (5 6 ))((10 (8 (4 9 )))(17 (16 (15 (13 (12 14 )))))))))(19 (21 (18 (20 (22 23 )))))))))*
(0 (2 (1 ((11 (7 ((3 (5 6 ))((10 (4 (8 9 )))(17 (16 ((12 13 )(14 15 ))))))))(19 (21 ((18 20 )(22 23 ))))))))*
(0 (2 (1 ((11 (7 ((3 (5 6 ))((10 (4 (8 9 )))(17 (16 (15 (14 (12 13 )))))))))(19 (18 (22 (23 (20 21 )))))))))*
(0 (2 (1 ((11 (7 ((3 (5 6 ))((10 (8 (4 9 )))(17 (16 (15 (13 (12 14 )))))))))(19 (18 (23 (22 (20 21 )))))))))*
(0 (2 (1 ((11 (7 ((3 (5 6 ))((10 (8 (4 9 )))(17 (16 ((12 13 )(14 15 ))))))))(19 (18 (23 (21 (20 22 )))))))))*
(0 (2 (1 ((11 (7 ((3 (5 6 ))((10 (4 (8 9 )))(17 (16 ((12 13 )(14 15 ))))))))(19 ((18 21 )(23 (20 22 ))))))))*
(0 (2 (1 ((11 (7 ((3 (5 6 ))((10 (8 (4 9 )))(17 (16 (15 (14 (12 13 )))))))))(19 ((18 20 )(22 (21 23 ))))))))*
(0 (2 (1 ((11 (7 ((3 (5 6 ))((10 (8 (4 9 )))(17 (16 (15 (13 (12 14 )))))))))(19 ((18 20 )(23 (21 22 ))))))))*
(0 (2 (1 ((11 (7 ((3 (5 6 ))((10 (4 (8 9 )))(17 (16 (15 (13 (12 14 )))))))))(19 (18 (22 (21 (20 23 )))))))))*
(0 (2 (1 ((11 (7 ((3 (5 6 ))((10 (4 (8 9 )))(17 (16 (15 (13 (12 14 )))))))))(19 (18 (21 (23 (20 22 )))))))))*
(0 (2 (1 ((11 (7 ((3 (5 6 ))((10 (4 (8 9 )))(17 (16 (15 (13 (12 14 )))))))))(19 ((21 (18 20 ))(22 23 )))))))*
(0 (2 (1 ((11 (7 ((3 (5 6 ))((8 (10 (4 9 )))(17 (16 (14 (15 (12 13 )))))))))(19 (18 (23 (21 (20 22 )))))))))*
(0 (2 (1 ((11 (7 ((3 (5 6 ))((10 (4 (8 9 )))(17 (16 ((12 13 )(14 15 ))))))))(19 (20 (18 (23 (21 22 )))))))))*
(0 (2 (1 ((11 (7 ((3 (5 6 ))((10 (8 (4 9 )))(17 (16 (15 (13 (12 14 )))))))))(19 ((20 (18 21 ))(22 23 )))))))*
(0 (2 (1 ((11 (7 ((3 (5 6 ))((8 (10 (4 9 )))(17 (16 (15 (13 (12 14 )))))))))(19 (21 ((18 20 )(22 23 ))))))))*
(0 (2 (1 ((11 (7 ((3 (5 6 ))((10 (8 (4 9 )))(17 (16 ((12 13 )(14 15 ))))))))(19 ((18 21 )(22 (20 23 ))))))))*
(0 (2 (1 ((11 (7 ((3 (5 6 ))((8 (10 (4 9 )))(17 (16 (15 (13 (12 14 )))))))))(19 (20 (18 (23 (21 22 )))))))))*
(0 (2 (1 ((11 (7 ((3 (5 6 ))((8 (10 (4 9 )))(17 (16 (15 (13 (12 14 )))))))))(19 (18 (20 (23 (21 22 )))))))))*
(0 (2 (1 ((11 (7 ((3 (5 6 ))((8 (10 (4 9 )))(17 (16 (15 (13 (12 14 )))))))))(19 (21 (18 (23 (20 22 )))))))))*
(0 (2 (1 ((11 (7 ((3 (5 6 ))((8 (10 (4 9 )))(17 (16 ((12 13 )(14 15 ))))))))(19 ((18 20 )(22 (21 23 ))))))))*
(0 (2 (1 ((11 (7 ((3 (5 6 ))((10 (4 (8 9 )))(17 (16 (15 (13 (12 14 )))))))))(19 ((18 20 )(23 (21 22 ))))))));
proc-;
```
